# Investigating the Conformations of a Family of [M_2_L_3_]^4+^ Helicates Using Single Crystal X-ray Diffraction

**DOI:** 10.3390/molecules28031404

**Published:** 2023-02-01

**Authors:** Matthew J. Wallis, Hyunsung Min, Leonard F. Lindoy, Feng Li

**Affiliations:** 1 School of Science, Western Sydney University, Locked Bag 1797, Penrith, NSW 2751, Australia; 2 School of Chemistry, University of Sydney, Camperdown, NSW 2006, Australia

**Keywords:** metallosupramolecular, helicate, transition metal, metalloligand

## Abstract

We present five new dinuclear triple helicate compounds of types [Mn_2_**L**_3_](ClO_4_)_4_, [Co_2_**L**_3_](BF_4_)_4_, [Ni_2_**L**_3_](BF_4_)_4_, [Cu_2_**L**_3_](BF_4_)_4_, and [Zn_2_**L**_3_](BF_4_)_4_, where **L** is a previously reported semi-rigid ligand incorporating two α-diimine primary donor groups and two secondary 4-pyridyl donor groups. All complexes have been characterized in both solution and the solid state. Single crystal X-ray diffraction studies were used to probe the variation in the respective helical structures as the coordinated metal ion was altered, including the effect on the orientations of the secondary binding domains. The influence of the metal ion size, the spin state in the case of Fe(II), and the presence of Jahn-Teller distortions on the overall helical structure has been investigated. These results form a basis for the design and construction of new large metallosupramolecular architectures which manifest properties associated with the constituent helical metalloligand units.

## 1. Introduction

Metallosupramolecular assemblies have received very considerable attention over recent decades, affording a multitude of properties that can be tailored to a range of applications [1,2,3,4,5,6]. In the quest for greater control over the characteristics of these architectures, the design of increasingly complicated supramolecular topologies is of considerable ongoing interest. A variety of synthetic strategies have been developed in this regard, including the use of the subcomponent self-assembly approach [7], the molecular library approach [8,9], the symmetry interaction approach [10], and the molecular paneling approach [11], to name a few.

Recently, our group has been interested in the metalloligand approach for constructing metallosupramolecular entities [12,13,14]. This method employs a coordination complex (or complexes) with secondary binding domains as building units for achieving larger self-assembled structures with both discrete [12,13,14] and continuous [15,16] architectures. The approach is to design ligands that form a complex with a primary metal while keeping additional (secondary) coordination donor sites free. The primary metal acts to direct the coordination vectors of the secondary binding sites so that they are aligned for coordination with secondary metal centers in forming the final structure [12,13,14,15,16,17,18,19,20,21]. Using the metalloligand approach, it is thus usually possible to transfer, at least in part, the physical properties of the metalloligand units, such as luminescence and various magnetic behaviors, to the larger final architecture.

To date, the majority of the metalloligands that have been reported are mononuclear, with higher nuclearity metalloligands being comparatively rare [22,23,24,25,26]. Currently, there are a few examples of dinuclear metalloligands [20,21,22], as well as helical metalloligands [26]. However, to the best of our knowledge, no dinuclear triple helicate metalloligands have so far been incorporated in the synthesis of larger supramolecular architectures, though potential candidates have been identified [27].

We have recently shown how the magnetic properties of a tripodal mononuclear Fe(II) metalloligand, originally in the high-spin state, undergoes a spin transition when a cubic cage is formed using Ni(II) as the secondary metal ion [20]. In contrast, the substitution of Pd(II) for Ni(II) resulted in the cage maintaining its Fe(II) centers high-spin [21]. In another study, we analyzed in some detail how the presence of a spin transition in a Fe(II) dinuclear triple helicate influenced the structure of the molecule, including the respective orientations of the secondary binding domains [28].

**Figure 1:**
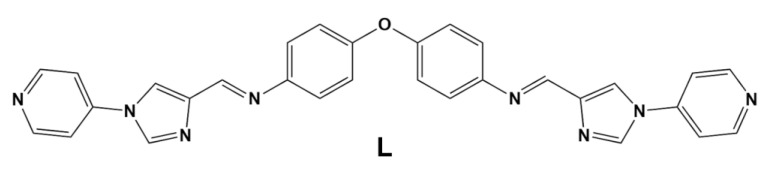
Structure of ligand **L**.

Herein we present five new dinuclear triple helicates of type [M_2_L_3_]X_4_, where M = Mn(II), Co(II), Ni(II), Cu(II) or Zn(II), **L** is a previously reported ligand (Figure 1) [28], and X is either BF_4_^−^ or ClO_4_^−^. For this series of helicates, we analyzed the effect that the choice of the primary metal ion has on the respective complex structures, including on the orientations of the secondary binding domains. In particular, SCXRD has been employed to probe the effect that the coordinated primary metal ion has on the respective helicate structures with respect to metal ion size, Fe(II) spin state, and the presence of Jahn-Teller distortions. This work represents a precursor study; the secondary coordinating sites of the helicate metalloligands will be later employed for the synthesis of larger metallosupramolecular architectures.

## 2. Results and Discussion

### 2.1. Synthesis and Characterisation

The reaction of the different M(II) salts (M = Mn, Co, Ni, Cu, and Zn) with previously reported **L** [28] in MeCN was followed by slow vapor diffusion of diethyl ether into the respective reaction mixtures to yield suitable crystals for SCXRD analysis in each case. The high-resolution electrospray ionization mass spectra (HR ESI-MS) of all complexes yielded *m*/*z* values corresponding to [M_2_**L**_3_(X)_2_]^2+^ (M = Mn, Co, Ni, Cu, and Zn, with X = BF_4_^-^ or ClO_4_^−^), [M_2_**L**_3_(X)]^3+^ and [M_2_**L**_3_]^4+^ (Figures S1–S5). The major peaks for four helicates (Co, Ni, Cu, and Zn) were observed at *m*/*z* values of 412.0855, 412.0855, 414.0676, and 415.0863, respectively, corresponding in each case to the 4+ species, with the *m*/*z* values for all four helicates being in good agreement with the simulated isotopic patterns (with the simulated peaks occurring at *m*/*z* values 412.1103, 412.1114, 414.1085, and 415.1083 for [Co_2_**L**_3_](BF_4_)_4_, [Ni_2_**L**_3_](BF_4_)_4_, [Cu_2_**L**_3_](BF_4_)_4_ and [Zn_2_**L**_3_](BF_4_)_4_ helicates respectively). In the case of [Mn_2_**L**_3_](ClO_4_)_4_, the major peak occurred at *m*/*z* 579.7500, which corresponds to the 3+ species and is in agreement with the simulated *m*/*z* value of 579.8025. The isotopic patterns for all other charge states are also in good agreement with their experimental values.

The SEM images of all complexes indicated that the above crystals undergo decay and cracking upon loss of their solvents (Figure S6). The EDS analyses confirmed the presence of the expected elements for all compounds.

### 2.2. Crystallography

**Figure 2:**
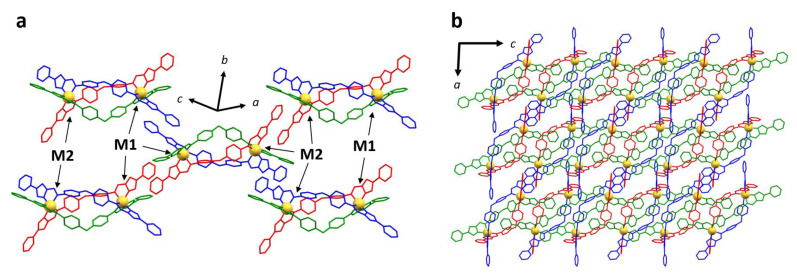
General packing schematic for all metal complexes in the present series, viewed (**a**) along the (−2, 1, −2) axis, (**b**) viewed along the *b*-axis. This particular image was generated using the [Co_2_**L**_3_](BF_4_)_4_ structure. Note that the same packing configuration is observed in all crystal structures discussed in this study. Colour scheme is ligand **A** (red), ligand **B** (blue), ligand **C** (green), metal atoms (orange). Hydrogen atoms, anions, and solvents have been omitted for clarity.

Synchrotron SCXRD confirmed the formation of all five helicates (Figure 2) with two metal centers (M = Mn, Co, Ni, Cu, and Zn) coordinated with three bis-bidentate ligands. [Mn_2_**L**_3_](ClO_4_)_4_, [Co_2_**L**_3_](BF_4_)_4_, [Ni_2_**L**_3_](BF_4_)_4_ and [Zn_2_**L**_3_](BF_4_)_4_ crystallized in *P*-1 space group with one helicate molecule, four tetrafluoroborate or perchlorate anions, four acetonitrile and one diethyl ether and zero or one water molecules in various occupancies present in the asymmetric unit. Solvent masks were applied in all four structures accounting for low-intensity electron density, most likely arising from disordered and low occupancy solvents in the structure. Each homochiral helicate (ΔΔ or ΛΛ) found in the asymmetric unit is related by inversion to its enantiomeric opposite. The coordination environment of the M(II) centers is composed of three imidazoleimine donor groups, with six nitrogens (three imine and three imidazole donors) coordinating to give a distorted octahedral arrangement.

All crystal structures display the same packing arrangement as that found previously for [Fe_2_**L**_3_](BF_4_)_4_ (Figure 2) [28]. The special case of [Cu_2_**L**_3_](BF_4_)_4_, while displaying the same packing motif, had a tripled unit cell volume (see below). The three bis-bidentate ligands (**A**, **B,** and **C**) in all helicate structures are associated with different sets of intermolecular interactions. Ligand **A** forms a continuous 1D chain, stabilized through hydrogen bonding between the pyridyl N atom and imidazole CH group, as well as π-π interactions between adjacent helicates. The section of ligand **B** that is proximal to metal 1 (M1) is also shown to participate in offset π-π interactions with an equivalent terminal group of a neighboring helicate of opposite chirality. The distal component of ligand **B** close to metal 2 (M2) does not engage in any π-π stacking due to the neighboring rings being in an unfavorable alignment, affected by the position of counterions. The pyridyl group of ligand **C** at the M2 end has no intermolecular contacts with adjacent helicates; however, the pyridyl group residing at the M1 end of the helicate lies close to the central O atom of ligand **A** from an adjacent helicate with opposite chirality.

**Figure 3:**
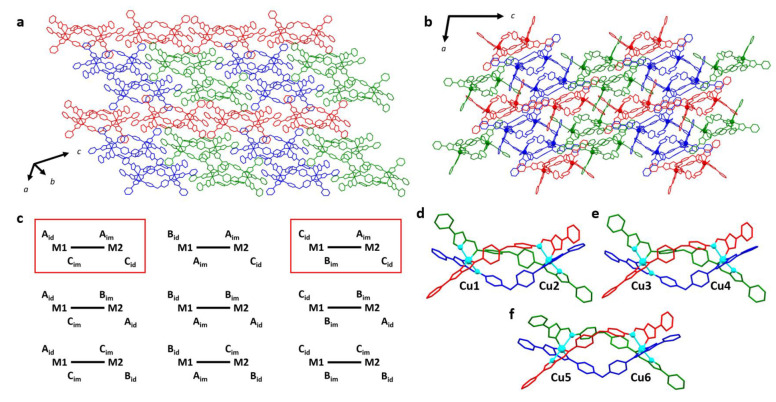
(**a**) Crystal packing for [Cu_2_**L**_3_](BF_4_)_4_ highlighting the crystallographically inequivalent helicates *Cu1-2* (red), *Cu3-4* (blue), and *Cu5-6* (green). Chains defined by ligand **A** run left-to-right in this projection. Adjacent *Cu3-4* and *Cu5-6* 1D chains differ by the chirality of the units comprising the chain. (**b**) The same structure is viewed along the crystallographic *b*-axis. Hydrogen atoms, anions, and solvents have been omitted for clarity. (**c**) Configurations of Jahn-Teller elongated axes, which are possible in the unsymmetric helicate units. The orientation of the elongated axis at each metal site is defined by the ligand donor atoms opposite to each other with the longest bond lengths. **A**, **B**, and **C** refer to the ligand associated with the donor atom, and subscripts “im” and “id” refer to the imidazole and imine N donors, respectively. Nine configurations are possible, and those present in [Cu_2_**L**_3_](BF_4_)_4_ are highlighted. *Cu1-2* and *Cu3-4* both exhibit the configuration of **C**_id_–M1–**B**_im_/**A**_im_–M2–**C**_id_ (top right), while *Cu5-6* bears the configuration **A**_id_–M1–**C**_im_/**A**_im_–M2–**C**_id_ (top left). The Jahn-Teller configurations of (**d**) *Cu1-2*, (**e**) *Cu3-4*, and (**f**) *Cu5-6* are shown with ligand **A** (red), ligand **B** (blue), and ligand **C** (green), while the Jahn-Teller elongated axes are highlighted in cyan.

The asymmetric unit for [Cu_2_**L**_3_](BF_4_)_4_ consists of three helicate molecules and twelve tetrafluoroborate anions. Positions of nineteen acetonitriles, four diethyl ethers, and one water molecule, with various occupancies, were found in the asymmetric unit. No residual voids required the implementation of a solvent mask. The same packing motif is present as found for the other crystal structures, despite the lattice being made up of three crystallographically inequivalent helicates (Figure 3). The three helicates in the asymmetric unit differ by their association with other helicate units in the lattice and the configuration of Jahn-Teller elongated axes in the coordination spheres. Each helicate has three non-equivalent ligands in the crystal structure, allowing the possibility for the Jahn-Teller elongated axis to occur in one of three orientations at each metal center. Since both metal centers are also inequivalent, there are nine possible configurations that might be brought about by Jahn-Teller elongations manifesting along different coordination axes of the two metal centers (Figure 3c), with two of these configurations expressed in the crystal structure (Figure 3c–f). In the discussion that follows, the distinct helicate cations will be referred to as *Cu1-2*, *Cu3-4*, and *Cu5-6*, with the numbers referring to the labels given to each Cu atom in the crystal structure (ligands **A, B** and **C** were, respectively, labeled as **D**, **E,** and **F** in *Cu3-4*, and **G**, **H,** and **I** in *Cu5-6*). *Cu1-2* forms connections through linkages (across inversion centers) along ligand **A** to enantiomeric opposite *Cu1-2* units, giving rise to 1D chains composed entirely of *Cu1-2* units. On the other hand, *Cu3-4* and *Cu5-6* comprise a different category of 1D chains, wherein *Cu3-4* and *Cu5-6* alternate along the 1D chain defined by ligand **A**; specifically, the repeating pattern of Cu atoms …*3-4*…*6-5*…*3-4*…*6-5*… describes the chains. Two distinct chains are found for the *Cu3-4* and *Cu5-6* pair, one for which *Cu3-4* is ΔΔ and connects to *Cu5-6* units, which are ΛΛ, and another where ΛΛ *Cu3-4* units connect to ΔΔ *Cu5-6* helicates (Figure 3a,b). Another key difference in the three inequivalent helicates in this structure is the configuration of Jahn-Teller elongated axes at each metal center (Figure 3c–f). The Jahn-Teller elongated axis of each metal will be described here with the general notation **I**N_id_-Cu#-**J**N_im_, where **I** and **J** refer to the ligands to which the relevant donor atoms belong, and the subscripts “im” and “id” refer to the imidazole and imine N donors respectively. In *Cu1-2*, the configuration expressed by **C**N_id_-Cu1-**B**N_im_/**A**N_im_-Cu2-**C**N_id_ is observed. The same configuration is observed in *Cu3-4*, although for *Cu5-6*, the Jahn-Teller elongation occurred according to **A**N_id_-Cu1-**C**N_im_/**A**N_im_-Cu2-**C**N_id_. In all three helicates, the even-numbered metal centers (which hold similar positions in the lattice) share the same Jahn-Teller configuration, while the elongated axis of the odd-numbered metal centers differed only at *Cu5*. In all coordination spheres, the Jahn-Teller elongated axis contains one of the ligand **C** coordination bonds, which may be related to the relative steric “freedom” of this ligand in the supramolecular arrangement, having no strong contacts with neighboring helicates.

The octahedral distortion parameters measuring the average length of coordination bonds and the sum of angular distortion values represented by Σ and Θ were calculated using OctaDist (Table 1) [29]. The largest metal ion in the series is Mn(II), which gave average coordinate bond lengths of 2.26 Å for both metal centers. The structures of [HS–HS] [Fe_2_**L**_3_](BF_4_)_4_, [Co_2_**L**_3_](BF_4_)_4_, [Ni_2_**L**_3_](BF_4_)_4_, and [Zn_2_**L**_3_](BF_4_)_4_ also showed consistency between coordinate bond lengths for M1 and M2, returning respective values (M1/M2) of 2.19/2.20, 2.15/2.15, 2.10/2.11, and 2.18/2.18 Å. The same pattern emerged from the three helicates of [Cu_2_**L**_3_](BF_4_)_4_, with the average coordinate bond distances being 2.16/2.16, 2.17/2.16, and 2.17/2.16 Å for *Cu1-2*, *Cu3-4*, and *Cu5-6*, respectively. This consistency is also reflected fairly well for the most part in the angular distortion values (Table 1, Figure S7). One key exception arises for [HS–LS] [Fe_2_**L**_3_](BF_4_)_4_, in which the LS state of Fe2 reduces the coordinate bond length to 2.00 Å, leading to a marked decrease in all the other octahedral distortion parameters. The Jahn-Teller elongated axes in [Cu_2_**L**_3_](BF_4_)_4_ led to high ζ values that fell in the range of 0.89–1.06 Å, whereas the highest ζ found in another structure (Zn2) was 0.36 Å (Figure S7c). The octahedral distortion parameters suggest that the larger size of the metal ion (as reflected by the average coordinate bond length) also corresponds to higher angular distortions. [Mn_2_**L**_3_](ClO_4_)_4_ bears the highest octahedral distortion values while [Ni_2_**L**_3_](BF_4_)_4_ and the LS Fe(II) centre observed in the [HS–LS] structure of [Fe_2_**L**_3_](BF_4_)_4_ return the lowest values.

**Figure 4:**
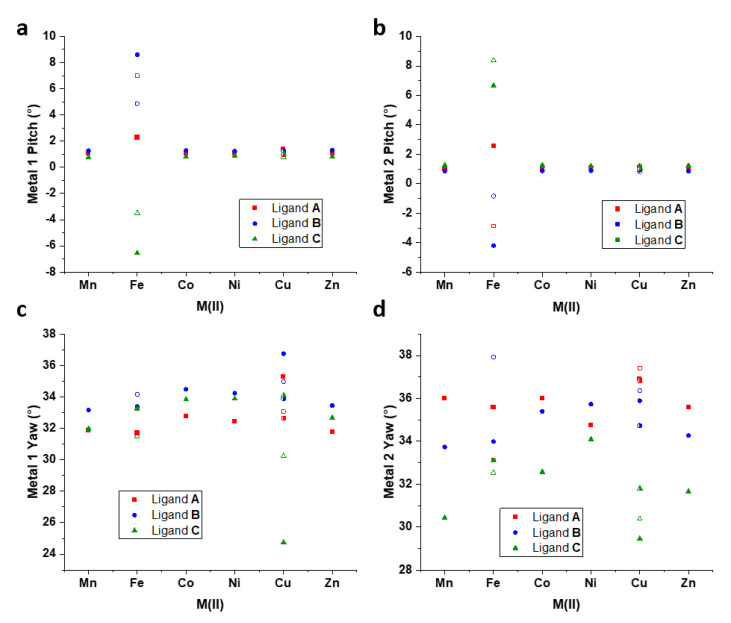
Pitch and Yaw for the [M_2_**L**_3_]^4+^ complexes (**a**) Pitch of chelate rings at M1, (**b**) Pitch of chelate rings at M2, (**c**) Yaw of chelate rings at M1, and (**d**) Yaw of chelate rings at M2. Solid shapes are used to represent [HS–HS] [Fe_2_**L**_3_](BF_4_)_4_ and *Cu1-2* of [Cu_2_**L**_3_](BF_4_)_4_, hollow shapes refer to [HS–LS] [Fe_2_**L**_3_](BF_4_)_4_ and *Cu3-4* of [Cu_2_**L**_3_](BF_4_)_4_, and half-colored shapes refer to *Cu5-6* of [Cu_2_**L**_3_](BF_4_)_4_.

As in the previous report discussing [Fe_2_**L**_3_](BF_4_)_4_ [28], the parameters pitch and yaw have been calculated for the present systems and describe the orientations of chelate groups relative to the intermetallic axis. Pitch describes the back-and-forth rocking orientation of a chelate ring, while yaw refers to the side-to-side twisting of the chelate group. Details for the calculation of these parameters are given in Supplementary Materials (S4). The pitch values show remarkable constancy across the present series of complexes, with the measured angles falling in the range of 0.8–1.3° (Figure 4a,b, Table S2). This is contrasted by the reported pitch values for both the [HS–HS] and [HS–LS] structures of [Fe_2_**L**_3_](BF_4_)_4_, for which the values varied from −6.6° to 8.6° between the two structures [28]. The latter is undoubtedly related to the effect of spin equilibrium on the [Fe_2_**L**_3_](BF_4_)_4_ structure, and it was shown that a decrease in pitch at Fe2 from 2.6 to −2.9° was associated with the full relaxation to the [HS-LS] state. Unexpectedly, the Jahn-Teller elongations in all three [Cu_2_**L**_3_](BF_4_)_4_ helicates had no significant impact on the pitch. In [Mn_2_**L**_3_](ClO_4_)_4_, [HS–HS] [Fe_2_**L**_3_](BF_4_)_4_, [Co_2_**L**_3_](BF_4_)_4_, [Ni_2_**L**_3_](BF_4_)_4_ and [Zn_2_**L**_3_](BF_4_)_4_, the yaw angles of ligands **A**, **B** and **C** follow the same general pattern at M1 in each structure. At this site, ligand **B** exhibits the greatest yaw, with the lowest associated with ligand **A** and a tight grouping of approximately 2° between maximum and minimum angles. At M2, the same structures displayed a wide spread of yaw angles, with the highest values found for ligand **A** (with the exception of [Ni_2_**L**_3_](BF_4_)_4_). The larger metal ions Mn(II) and Zn(II) adopted a wider spread of yaw values than the smaller ions, suggesting that the larger metal ions allow for a greater degree of chelate ring flexibility, which coincides well with the higher octahedral distortion values obtained for these ions. The [HS–LS] [Fe_2_**L**_3_](BF_4_)_4_ structure, as well as all three [Cu_2_**L**_3_](BF_4_)_4_ helicates, showed a wider spread of yaw values at both metal centers. This demonstrates that the varying degrees of coordination sphere distortion associated with the ions’ electron configurations contribute to the pliability of the chelate groups with respect to the helical axis.

**Figure 5:**
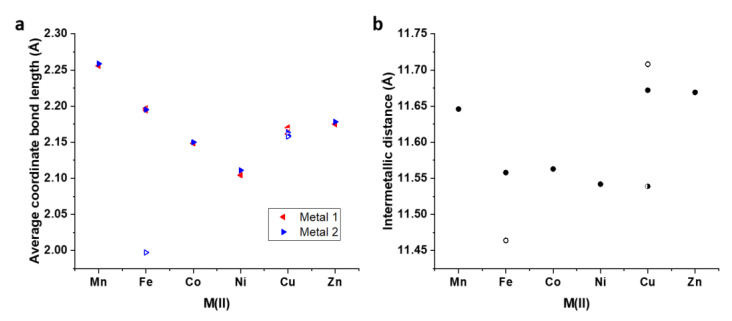
Plots of (**a**) Average coordinate bond length and (**b**) Intermetallic distances. Solid shapes are used to represent [HS–HS] [Fe_2_**L**_3_](BF_4_)_4_ and *Cu1-2* of [Cu_2_**L**_3_](BF_4_)_4_, hollow shapes refer to [HS-LS] [Fe_2_**L**_3_](BF_4_)_4_ and *Cu3-4* of [Cu_2_**L**_3_](BF_4_)_4_, and half-colored shapes refer to *Cu5-6* of [Cu_2_**L**_3_](BF_4_)_4_.

The larger metal ions in [Mn_2_**L**_3_](ClO_4_)_4_ and [Zn_2_**L**_3_](BF_4_)_4_ yielded intermetallic distances of 11.65 and 11.67 Å, respectively, while the smaller ions in [HS–HS] [Fe_2_**L**_3_](BF_4_)_4_, [Co_2_**L**_3_](BF_4_)_4_ and [Ni_2_**L**_3_](BF_4_)_4_ gave rise to smaller values of 11.56, 11.56, and 11.54 Å, respectively (Figure 5). The shortest distance was obtained for the [HS–LS] conformer of [Fe_2_**L**_3_](BF_4_)_4_, for which the shrinking of the coordination sphere of Fe2 resulted in the shortening of this helicate to 11.47 Å. On the other hand, the Jahn-Teller distortions present in the [Cu_2_**L**_3_](BF_4_)_4_ helicates led to intermetallic distances of 11.67, 11.71, and 11.54 Å for *Cu1-2*, *Cu3-4*, and *Cu5-6*, respectively, with the longer two distances arising for the two helicates of Jahn-Teller configuration **C**_id_–Cu1–**B**_im_/**A**_im_–Cu2–**C**_id_ (Figure 3c–f).

**Figure 6:**
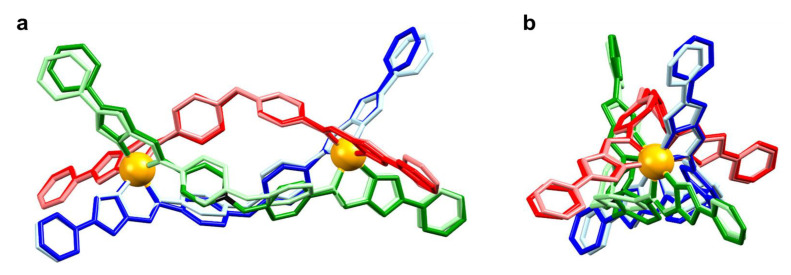
Overlay of [Mn_2_**L**_3_]^4+^ (darker colors) and [Ni_2_**L**_3_]^4+^ (lighter colors). Color scheme, ligand **A** (red), ligand **B** (blue), ligand **C** (green). (**a**) Shows the molecules perpendicular to the pseudo-threefold axis with M1 on the left and M2 on the right. (**b**) The same overlay looks down the pseudo-threefold axis, with M2 projected out of the page and M1 hidden behind M2. Hydrogen atoms, anions, and solvents have been omitted for clarity.

Since the structure connecting the coordinating imidazoleimine domain and the secondary pyridyl group is rigid, the orientation of each secondary binding domain is linked directly to the orientation of the conjoined chelate ring. As such, the pitch and yaw associated with each chelate ring give rise to a complementary orientation of the secondary bonding axis. Parameters describing the orientation of secondary bonding axes have been employed to investigate the effect of the size and electronic configuration of the primary metal ion, as occurred in our previous work [20,21,28]. The “secondary bond axis” (2BA) parameter describes the angular deviation of the secondary coordination axis (as defined by the axis between N and opposite C in the pyridyl group) from the intermetallic axis [28]. On the other hand, the “mutual bond axis angle” (MBA) describes the orientation of secondary coordination axes (proximal to one metal site) to each other [20,21]. These parameters provide complementary information and may be used to predict the derivative supramolecular topologies that might be targeted by these metalloligands. To exemplify the structural conformations which are highlighted by the parameters 2BA, the helicate structures of [Mn_2_**L**_3_](ClO_4_)_4_ and [Ni_2_**L**_3_](BF_4_)_4_ have been overlaid (Figure 6). The terminal pyridyl groups, which display consistent positions, bear similar values for 2BA. On the other hand, the notable differences in 2BA at the M1 end of ligands **C** and **B**, as well as the differences in values at M2 for ligands **A** and **B,** give rise to poorly overlapped groups in the figure, with larger differences arising from larger differences in the parameter values.

**Figure 7:**
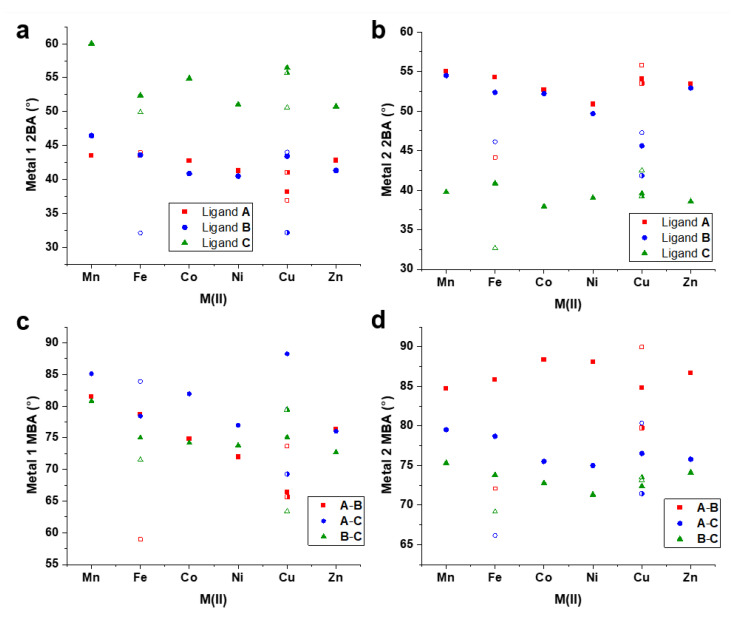
(**a**) 2BA angles at the M1 end of each helicate, (**b**) 2BA angles at the M2 end of each helicate, (**c**) MBA angles at the M1 end of each helicate, (**d**) MBA angles at the M2 end of each helicate. Solid shapes are used to represent [HS–HS] [Fe_2_**L**_3_](BF_4_)_4_ and *Cu1-2* of [Cu_2_**L**_3_](BF_4_)_4_, hollow shapes refer to [HS-LS] [Fe_2_**L**_3_](BF_4_)_4_ and *Cu3-4* of [Cu_2_**L**_3_](BF_4_)_4_, and half-colored shapes refer to *Cu5-6* of [Cu_2_**L**_3_](BF_4_)_4_.

In metalloligands [Mn_2_**L**_3_](ClO_4_)_4_, [Co_2_**L**_3_](BF_4_)_4_, [Ni_2_**L**_3_](BF_4_)_4_ [Zn_2_**L**_3_](BF_4_)_4_ and the [HS-LS] structure of [Fe_2_**L**_3_](BF_4_)_4_, the values of ligand **C** 2BA at M1 fall in the range of 51.00°–60.0°, systematically higher than the tightly grouped values found for ligands **A** and **B** which gave values in the range 40.5°–46.4° (Figure 7a). This may reflect the steric constraint imposed on the terminal pyridyl moieties of the **A** and **B** ligands at this end of the helicate in the observed crystal structures. The opposite trend is observed for these metalloligands at the M2 end of the helicate, with ligand **C** returning low 2BA values of 38.6°–40.9°, while the higher values for ligands **A** and **B** are grouped closely in the range 49.7°–55.0° (Figure 7b). In the [HS-LS] structure of [Fe_2_**L**_3_](BF_4_)_4_ and the Jahn-Teller distorted helicates found in [Cu_2_**L**_3_](BF_4_)_4_, the values for **C** 2BA were systematically highest at M1 and lowest at M2, similar to the other structures, but the grouping of the values associated with ligands **A** and **B** at both ends of the helicate are much looser than in the helicates which have no spin-state differences or Jahn-Teller distortions. The MBA values obtained at the M1 end of the molecule (Figure 7c) correlate with the size of the primary metal (Figure 5a), except in the cases associated with differentiated spin states and Jahn-Teller distortions. At the M1 end of the helicate, the MBA values were tightly grouped, while the M2 end gave rise to a wide spread of MBA values, with the angle between the secondary coordination axes of ligands **A** and **B** being systematically larger than the other two by some margin (Figure 7d).

The contrasting trends at either end of the helicates in yaw, as well as 2BA and MBA, are likely related to the intermolecular contacts present in the crystal packing arrangement, which are consistent across the series. The intermolecular interactions present in the terminals of ligands **A** and **B** at M1 contribute some constraint on the helicate, and the strain imparted to the helicate by these contacts may be compensated by complementary reconfigurations at the other end of the helicate. The wide range of angular values describing secondary coordination axes for each helicate suggests that the ligands may have enough flexibility to access orientations necessary for the formation of multiple topologies. Thus, careful selection of secondary metal ions may lead the way to the synthesis of a variety of larger derivative structures.

## 3. Materials and Methods

### 3.1. General Synthetic Procedure

A solution of the required metal salt (MX_2_; M = Mn(II), Co(II), Ni(II), Cu(II), Zn(II); X = BF_4_^−^. ClO_4_^−^) (2 eq.) in acetonitrile (MeCN) was added dropwise to a suspension of **L** (3 eq.) in MeCN. In each case, the reaction mixture was refluxed for 1 h with stirring, and the mixture was gravity filtered. The filtrates were diffused slowly with diethyl ether, and the resulting crystals were separated and air-dried before analysis.

[Mn_2_**L**_3_](ClO_4_)_4_: Manganese(II) perchlorate hydrate (111 mg, 0.27 mmol) in 30 mL of MeCN was added to **L** (200 mg, 0.39 mmol) in 20 mL of MeCN. Colorless crystals of [Mn_2_**L**_3_](ClO_4_)_4_ were obtained, yielding: 42 mg, 15.6%. HR ESI-MS (positive ion detection, MeCN): *m*/*z* (calc, exp) = [Mn_2_**L**_3_]^4+^; 410.1128, 410.0569; [Mn_2_**L**_3_(ClO_4_)]^3+^; 579.8025, 579.7500; [Mn_2_**L**_3_(ClO_4_)_2_]^2+^; 920.1740, 920.1578.

[Co_2_**L**_3_](BF_4_)_4_: Cobalt(II) tetrafluoroborate hexahydrate (91 mg, 0.27 mmol) in 30 mL of MeCN was added to **L** (200 mg, 0.39 mmol) in 20 mL of MeCN. Orange crystals of [Co_2_**L**_3_](BF_4_)_4_ were obtained, yielding: 70 mg, 26.9%. HR ESI-MS (positive ion detection, MeCN): *m*/*z* (calc, exp) = [Co_2_**L**_3_]^4+^; 412.1103, 412.0855; [Co_2_**L**_3_(BF_4_)]^3+^; 578.4800, 578.4698; [Co_2_**L**_3_(BF_4_)_2_]^2+^; 911.2272, 911.2087.

[Ni_2_**L**_3_](BF_4_)_4_: Nickel(II) tetrafluoroborate hydrate (91 mg, 0.27 mmol) in 30 mL of MeCN was added to **L** (200 mg, 0.39 mmol) in 20 mL of MeCN. Pale yellow crystals of [Ni_2_**L**_3_](BF_4_)_4_ were obtained, yielding: 85 mg, 32.4%. HR ESI-MS (positive ion detection, MeCN): *m*/*z* (calc, exp) = [Ni_2_**L**_3_]^4+^; 412.1114, 412.0855, [Ni_2_**L**_3_(BF_4_)]^3+^; 578.4867, 578.4698; [Ni_2_**L**_3_(BF_4_)_2_]^2+^; 911.2201, 911.2087.

[Cu_2_**L**_3_](BF_4_)_4_: Copper(II) tetrafluoroborate hydrate (64 mg, 0.27 mmol) in 30 mL of MeCN was added to **L** (200 mg, 0.39 mmol) in 20 mL of MeCN. Green crystals of [Cu_2_**L**_3_](BF_4_)_4_ were obtained, yielding: 82 mg, 31.1%. HR ESI-MS (positive ion detection, MeCN): *m*/*z* (calc, exp) = [Cu_2_**L**_3_]^4+^; 414.1085, 414.0676; [Cu_2_**L**_3_(BF_4_)]^3+^; 581.8162, 581.7798; [Cu_2_**L**_3_(BF_4_)_2_]^2+^; 916.2222, 916.2050.

[Zn_2_**L**_3_](BF_4_)_4_: Zinc(II) tetrafluoroborate hydrate (69 mg, 0.27 mmol) in 30 mL of MeCN was added to **L** (200 mg, 0.39 mmol) in 20 mL of MeCN. Colorless opaque crystals of [Zn_2_**L**_3_](BF_4_)_4_ were obtained, yielding: 78 mg, 29.8%. HR ESI-MS (positive ion detection, MeCN): *m*/*z* (calc, exp) = [Zn_2_**L**_3_]^4+^; 415.1083, 415.0863; [Zn_2_**L**_3_(BF_4_)]^3+^; 583.1466, 583.1248; [Zn_2_**L**_3_(BF_4_)_2_]^2+^; 918.2217, 918.1996.

### 3.2. Physical Measurements

Scanning Electron Microscopy and Energy Dispersive Spectroscopy (SEM-EDS) spot analysis were carried out using a Phenom XL in a low vacuum with a chamber pressure of 60 Pa and an accelerating voltage of 15 kV. All samples were mounted to an aluminum stub with double-sided conductive carbon tape. Images were taken uncoated, and spot EDS analysis was carried out using Phenom Element Identification (version 3.8.4.0) with a silicon drift detector (SDD). High-resolution electrospray ionization mass spectroscopy (HR ESI-MS) experiments were conducted using a Waters Xevo QToF mass spectrometer (Waters, Milford, MA, USA) operating in positive ion mode. The samples were dissolved in acetonitrile and infused directly into the ESI source via a syringe.

### 3.3. SCXRD

All single crystal data were collected employing the MX1 beamline at the Australian Synchrotron using silicon double crystal monochromatic radiation (λ = 0.71073 Å) [30]. For each structure, two collections were taken, each consisting of a sweep through θ of 360° but differing by the setting of κ to 0° or 180°. XDS software (version Jan 10, 2022) [31] was used for data integration, processing, scaling, and the merging of raw datasets. Absorption corrections were then applied at the synchrotron using SADABS (version 2.05) [32,33]. Structures were solved with ShelXT (version March 2018) [34] using the intrinsic phasing method. Refinement details are presented below. Crystallographic data in CIF format have been deposited at the Cambridge Crystallographic Data Centre with CCDC nos. 2,224,285–2,224,289. They are available free of charge from the Cambridge Crystallographic Data Centre, 12 Union Road, Cambridge CB2 1 EZ, UK; fax: (+44) 1223-336-033; or e-mail: deposit@ccdc.cam.ac.uk.

### 3.4. Refinement Details

Full-matrix least-squares refinements were carried out using a suite of SHELX programs (version March 2018) [35,36] via the Olex2 interface [37]. Several structures exhibited orientational disorder in the helicate molecule, particularly in the linker segments of some ligands, terminal pyridine groups, and in chelate rings in rare cases. The restraints DFIX, DANG, SADI, FLAT, and RIGU and the constraint EADP were implemented where appropriate to model the disorder in various moieties, and where the electron density could not be satisfactorily modeled using one part, linker and pyridine groups were split and refined against free variables. Counter ions and solvents also exhibited disorder in some cases, which was addressed using the restraints SADI, DFIX, DANG, and RIGU, and the constraint EADP. In particular cases, solvents and anions were modeled using idealized rigid bodies [38]. Some groups were not able to be modeled anisotropically due to unreasonable anisotropic displacement parameters or inhibiting the shift convergence to zero, so they were left isotropic. Solvent masks were applied for [Mn_2_**L**_3_](ClO_4_)_4_, [Co_2_**L**_3_](BF_4_)_4_, [Ni_2_**L**_3_](BF_4_)_4_, and [Zn_2_**L**_3_](BF_4_)_4_ to account for residual electron density, which could not be modeled, arising from an unknown contribution of solvent molecules in disordered arrangements. Hydrogen atoms were fixed in idealized positions using a riding model but were removed in some cases to allow the convergence of shift towards zero.

## 4. Conclusions

In this report, we present five new M(II) dinuclear helicates [Mn_2_**L**_3_](ClO_4_)_4_, [Co_2_**L**_3_](BF_4_)_4_, [Ni_2_**L**_3_](BF_4_)_4_, [Cu_2_**L**_3_](BF_4_)_4_, and [Zn_2_**L**_3_](BF_4_)_4_ which have been characterized by ESI-MS, SEM-EDS, and SCXRD. All five helicates crystallized in the *P*-1 space group with both enantiomers Δ and Λ present in the crystal lattice. All helicates were shown to have a similar packing motif. Changing the metal centers demonstrated that the larger M(II) ions resulted in higher distortion values, with the exception of the [HS-LS] [Fe_2_**L**_3_](BF_4_)_4_ and the Jahn-Teller distorted [Cu_2_**L**_3_](BF_4_)_4_ helicates. Clearly, the coordination environment of the metal centers in each helicate contributes to the orientation of bis-chelating groups and hence also to the orientation of the secondary binding domains. We propose that the detailed analysis of the structural features of the present series of dinuclear helicates provides a foundation for future studies involving the design and synthesis of new large metallosupramolecular architectures that exhibit properties derived from the constituent helical metalloligand units. Ongoing studies of this type will be undertaken by our group in the future.

## Figures and Tables

**Table 1:** Octahedral distortion parameters and intermetallic distances for [M_2_**L**_3_]X_4_.

Compounds	Temperature (K)	Average M(II)-N Distance (Å)	ζ (Å)	Σ (˚)	Θ (˚)	Intermetallic Distances (Å) ^a^
[Mn_2_L_3_](ClO_4_)_4_	100	Mn1: 2.26, Mn2: 2.26	Mn1: 0.20, Mn2: 0.27	Mn1: 98.4, Mn2: 101.5	Mn1: 313.0, Mn2: 347.8	11.65
[Fe_2_L_3_](BF_4_)_4_ [HS-LS] [28]	100	Fe1:2.20, Fe2: 2.00	Fe1: 0.20 Fe2: 0.10	Fe1: 83.4, Fe2: 58.3	Fe1: 264.5, Fe2: 189.7	11.46
[Fe_2_L_3_](BF_4_)_4_ [HS–HS] [28]	250	Fe1: 2.19, Fe2: 2.20	Fe1: 0.22, Fe2: 0.25	Fe1: 89.1, Fe2: 84.9	Fe1: 278.2, Fe2: 286.5	11.56
[Co_2_L_3_](BF_4_)_4_	100	Co1: 2.15, Co2: 2.15	Co1: 0.21, Co2: 0.23	Co1: 80.7, Co2: 78.9	Co1: 252.6, Co2: 278.0	11.56
[Ni_2_L_3_](BF_4_)_4_	100	Ni1: 2.10, Ni2: 2.11	Ni1: 0.23, Ni2: 0.20	Ni1: 68.2, Ni2: 70.2	Ni1: 218.9, Ni2: 233.7	11.54
[Cu_2_L_3_](BF_4_)_4_ (*Cu1-2*)	100 **^b^**	Cu1: 2.16, Cu2: 2.16	Cu1: 0.95, Cu2: 1.03	Cu1: 73.9, Cu2: 94.6	Cu1: 257.1, Cu2: 307.4	11.67
[Cu_2_L_3_](BF_4_)_4_ (*Cu3-4*)		Cu3: 2.17, Cu4: 2.16	Cu3: 0.99, Cu4: 1.06	Cu3: 74.2, Cu4: 93.4	Cu3: 253.8, Cu4: 311.4	11.71
[Cu_2_L_3_](BF_4_)_4_ (*Cu5-6*)		Cu5: 2.17, Cu6: 2.16	Cu5: 1.03, Cu6: 0.89	Cu5: 82.3, Cu6: 86.4	Cu5: 255.8, Cu6: 256.0	11.54
[Zn_2_L_3_](BF_4_)_4_	100	Zn1: 2.18, Zn2: 2.18	Zn1: 0.31, Zn2: 0.36	Zn1: 84.9, Zn2: 85.1	Zn1: 262.0, Zn2: 287.1	11.67

**^a^** Intramolecular metal distance along the pseudo threefold axis of the helicate structure. **^b^** Three crystallographically distinct [Cu_2_**L**_3_]^4+^ helicates are from the same crystal structure.

## Data Availability

Additional data are available in the Supplementary Materials for this paper.

## Supplementary Materials

The following supporting information can be downloaded at: https://www.mdpi.com/article/10.3390/molecules28031404/s1, Figure S1: HR ESI-MS spectra for [Zn_2_**L**_3_](BF_4_)_4_ in acetonitrile. Inset shows the isotopic pattern for [Zn_2_**L**_3_]^4+^ (bottom) with simulated pattern (top); Figure S2: HR ESI-MS spectra for [Cu_2_**L**_3_](BF_4_)_4_ in acetonitrile. Inset shows the isotopic pattern for [Cu_2_**L**_3_]^4+^ (bottom) with simulated pattern (top); Figure S3: HR ESI-MS spectra for [Ni_2_**L**_3_](BF_4_)_4_ in acetonitrile. Inset shows the isotopic pattern for [Ni_2_**L**_3_]^4+^ (bottom) with simulated pattern (top); Figure S4: HR ESI-MS spectra for [Mn_2_**L**_3_](ClO_4_)_4_ in acetonitrile. Inset shows the isotopic pattern for [Mn_2_L_3_(ClO_4_)]^3+^ (bottom) with simulated pattern (top); Figure S5: HR ESI-MS spectra for [Co_2_**L**_3_](BF_4_)_4_ in acetonitrile. Inset shows the isotopic pattern for [Co_2_**L**_3_]^4+^ (bottom) with simulated pattern (top); Figure S6: A backscattering SEM image (left) and corresponding EDS analysis (right) for (a) [Ni_2_**L**_3_](BF_4_)_4_; (b) [Zn_2_**L**_3_](BF_4_)_4_; (c) [Cu_2_**L**_3_](BF_4_)_4_; (d) [Co_2_**L**_3_](BF_4_)_4_ and (e) [Mn_2_**L**_3_](ClO_4_)_4_; Figure S7: Octahedral distortion parameters. (a) Σ; (b) Θ; (c) ζ. Solid shapes are used to represent [HS-HS] [Fe_2_**L**_3_](BF_4_)_4_ and *Cu1-2* of [Cu_2_**L**_3_](BF_4_)_4_, hollow shapes refer to [HS-LS] [Fe_2_**L**_3_](BF_4_)_4_ and *Cu3-4* of [Cu_2_**L**_3_](BF_4_)_4_, and half-coloured shapes refer to *Cu5-6* of [Cu_2_**L**_3_](BF_4_)_4_; Table S1: Crystallographic data table for compounds presented in this study; Table S2: Selected geometric parameters describing structural orientation information for the dinuclear triple helicates; S4: Chelate ring angles.

## Sample Availability

Samples of the compounds are not available from the authors.
