# Supplementary material for: Investigating the Conformations of a Family of [M2L3]4+ Helicates Using Single Crystal X-ray Diffraction
Source: Molecules. 2023 Feb 1;28(3):1404. doi: 10.3390/molecules28031404 (PMC9921459; doi:10.3390/molecules28031404)
Supplement: Supplementary file 1 [file molecules-28-01404-s001.zip › Final cifs/2224285.pdf]

No syntax errors found.
Please wait while processing ....

CIF dictionary
Interpreting this report

Datablock: mn

Bond precision:	C-C = 0.0094 A		Wavelength=0.71073
Cell:	a=16.060(3)	b=18.190(4)	c=20.480(4)
	alpha=79.38(3)	beta=88.85(3)	gamma=73.39(3)
Temperature:	100 K		

	Calculated	Reported
Volume	5632(2)	5632(2)
Space group	P -1	P -1
Hall group	-P 1	-P 1
Moiety formula	2(C90 H65 Mn2 N24 O3), 8(Cl O4), 3(C4 O), 7(C2 H3 N), 2(C2 N) [	C90 H66 Mn2 N24 O3, 4(Cl O4), C4 H10 O, 4.5(C2 H3 N), 0.5(C4 O)
Sum formula	C210 H151 Cl8 Mn4 N57 O41 [+ solvent]	C105 H75.50 Cl4 Mn2 N28.50 O20.50
Mr	4632.24	2316.11
Dx,g cm-3	1.366	1.366
Z	1	2
Mu (mm-1)	0.399	0.399
F000	2374.0	2374.0
F000'	2377.48	
h,k,lmax	18,21,24	18,21,24
Nref	19195	18961
Tmin,Tmax	0.923,0.992	
Tmin'	0.923	

Correction method= Not given

Data completeness= 0.988 Theta(max)= 24.712

R(reflections)= 0.1100(14564) wR2(reflections)= 0.3776(18961)

S = 1.671 Npar= 1862

The following ALERTS were generated. Each ALERT has the format
test-name_ALERT_alert-type_alert-level.
Click on the hyperlinks for more details of the test.

Alert level A

PLAT201_ALERT_2_A Isotropic non-H Atoms in Main Residue(s) .... 11 Report
N1C N2C C1C C2C C3C etc.

Author Response: Weak high-angle reflections result in high Rint. This affects refineme parameters wR2, lowers bond precision and probably does not describe anisotropic displacements correctly. Orientational disorder in the helicate may give rise to some reflections which are not compatible with the assignment of Bravais lattice Orientational disorder in the terminal pyridine of ligand C and the associated chelating imidazoleimine moiety could not be satisfactorily modelled with anisotropic displacement parameters.

Alert level B

PLAT084_ALERT_3_B High wR2 Value (i.e. > 0.25) .... 0.38 Report

Author Response: Weak high-angle reflections result in high Rint. This affects refineme parameters wR2, lowers bond precision and probably does not describe anisotropic displacements correctly. Orientational disorder in the helicate may give rise to some reflections which are not compatible with the assignment of Bravais lattice

PLAT315_ALERT_2_B Singly Bonded Carbon Detected (H-atoms Missing). C1ET Check

Author Response: Orientational disorder in some solvent molecules prevented anisotropic modelling, and in some cases hydrogen atoms were not fixed to allow the shift to converge to zero.

PLAT315_ALERT_2_B Singly Bonded Carbon Detected (H-atoms Missing). C4ET Check
PLAT934_ALERT_3_B Number of (Iobs-Icalc)/Sigma(W) > 10 Outliers .. 4 Check

Author Response: Weak high-angle reflections result in high Rint. This affects refineme parameters wR2, lowers bond precision and probably does not describe anisotropic

displacements correctly. Orientational disorder in the helicate may give rise to some reflections which are not compatible with the assignment of Bravais lattice

---

Alert level C

```
THETM01_ALERT_3_C  The value of sine(theta_max)/wavelength is less than 0.590
            Calculated sin(theta_max)/wavelength =    0.5882
PLAT082_ALERT_2_C High R1 Value ................................       0.11 Report
PLAT202_ALERT_3_C Isotropic non-H Atoms in Anion/Solvent .........       13 Check
            Cl3G    O9G     O10G    O11G    O12G    O1ET             etc.
PLAT220_ALERT_2_C NonSolvent   Resd 1  C   Ueq(max)/Ueq(min) Range       3.3 Ratio
PLAT234_ALERT_4_C Large Hirshfeld Difference O1D      --C16D     .      0.18 Ang.
PLAT241_ALERT_2_C High   'MainMol' Ueq as Compared to Neighbors of       N4A Check
And 3 other PLAT241 Alerts
More ...
PLAT242_ALERT_2_C Low    'MainMol' Ueq as Compared to Neighbors of      C28C Check
PLAT244_ALERT_4_C Low    'Solvent' Ueq as Compared to Neighbors of      C3ET Check
And 2 other PLAT244 Alerts
More ...
PLAT260_ALERT_2_C Large Average Ueq of Residue Including      Cl1G      0.159 Check
And 5 other PLAT260 Alerts
More ...
PLAT334_ALERT_2_C Small <C-C> Benzene Dist.   C16E     -C21E     .      1.37 Ang.
PLAT341_ALERT_3_C Low Bond Precision on  C-C Bonds ..............   0.00936 Ang.
PLAT906_ALERT_3_C Large K Value in the Analysis of Variance ......     4.452 Check
PLAT911_ALERT_3_C Missing FCF Refl Between Thmin & STh/L=    0.588       234 Report
PLAT918_ALERT_3_C Reflection(s) with I(obs) much Smaller I(calc) .        20 Check
PLAT922_ALERT_1_C wR2 in the CIF and FCF Differ by ..............   -0.0011 Check
PLAT975_ALERT_2_C Check Calcd Resid. Dens.  0.91Ang From C1ET    .      0.77 eA-3
```

Alert level G

```
FORMU01_ALERT_1_G  There is a discrepancy between the atom counts in the
            _chemical_formula_sum and _chemical_formula_moiety. This is
            usually due to the moiety formula being in the wrong format.
            Atom count from _chemical_formula_sum:   C105 H75.5 Cl4 Mn2 N28.5 O20.
            Atom count from _chemical_formula_moiety:C105 H89.5 Cl4 Mn2 N28.5 O20.
PLAT002_ALERT_2_G Number of Distance or Angle Restraints on AtSite       178 Note
PLAT042_ALERT_1_G Calc. and Reported MoietyFormula Strings  Differ    Please Check
PLAT045_ALERT_1_G Calculated and Reported Z Differ by a Factor ...     0.500 Check
PLAT072_ALERT_2_G SHELXL First  Parameter in WGHT  Unusually Large      0.20 Report
PLAT154_ALERT_1_G The s.u.'s on the Cell Angles are Equal ..(Note)      0.03 Degree
PLAT171_ALERT_4_G The CIF-Embedded .res File Contains EADP Records         1 Report
PLAT172_ALERT_4_G The CIF-Embedded .res File Contains DFIX Records        17 Report
PLAT173_ALERT_4_G The CIF-Embedded .res File Contains DANG Records        10 Report
PLAT174_ALERT_4_G The CIF-Embedded .res File Contains FLAT Records        28 Report
PLAT176_ALERT_4_G The CIF-Embedded .res File Contains SADI Records       213 Report
PLAT180_ALERT_4_G Check Cell Rounding: # of Values Ending with 0 =         3 Note
PLAT187_ALERT_4_G The CIF-Embedded .res File Contains RIGU Records        30 Report
PLAT191_ALERT_3_G A Non-default SADI Restraint Value has been used    0.0400 Report
And 100 other PLAT191 Alerts
More ...
PLAT244_ALERT_4_G Low    'Solvent' Ueq as Compared to Neighbors of      Cl1G Check
PLAT244_ALERT_4_G Low    'Solvent' Ueq as Compared to Neighbors of      Cl2G Check
PLAT300_ALERT_4_G Atom Site Occupancy of O1EV       Constrained at       0.5 Check
And 10 other PLAT300 Alerts
More ...
PLAT301_ALERT_3_G Main Residue  Disorder .............(Resd  1  )        48% Note
PLAT302_ALERT_4_G Anion/Solvent/Minor-Residue Disorder (Resd  4  )      100% Note
And 9 other PLAT302 Alerts
More ...
PLAT304_ALERT_4_G Non-Integer Number of Atoms in ..... (Resd  5  )      3.40 Check
And 6 other PLAT304 Alerts
More ...
PLAT315_ALERT_2_G Singly Bonded Carbon Detected (H-atoms Missing).      C1EV Check
```

Author Response: Orientational disorder in some solvent molecules prevented anisotropic modelling, and in some cases hydrogen atoms were not fixed to allow the shift to converge to zero.

```
And 3 other PLAT315 Alerts
More ...
PLAT343_ALERT_2_G Unusual sp?      Angle Range in Main Residue for       C9A Check
PLAT344_ALERT_2_G Unusual sp?       Angle Range in Solvent/Ion for      C1ET Check
And 3 other PLAT344 Alerts
More ...
PLAT410_ALERT_2_G Short Intra H...H Contact  H24A     ..H27D     .      1.93 Ang.
                                                  x,y,z  =      1_555 Check
```

```
And 2 other PLAT410 Alerts
More ...
PLAT432_ALERT_2_G Short Inter X...Y Contact  O14G     ..C22B     .       3.01 Ang.
                                                     x,y,z  =     1_555 Check
PLAT432_ALERT_2_G Short Inter X...Y Contact  O1F      ..C24C     .       2.98 Ang.
                                                 1-x,-y,2-z  =     2_657 Check
And 2 other PLAT432 Alerts
More ...
                                                   -1+x,y,z  =     1_455 Check
PLAT432_ALERT_2_G Short Inter X...Y Contact  C4ET     ..C1S3     .       2.82 Ang.
                                                   -1+x,y,z  =     1_455 Check
PLAT432_ALERT_2_G Short Inter X...Y Contact  C2S3     ..C27C     .       3.11 Ang.
                                                1-x,1-y,2-z  =     2_667 Check
PLAT605_ALERT_4_G Largest Solvent Accessible VOID in the Structure        84 A**3
PLAT720_ALERT_4_G Number of Unusual/Non-Standard Labels .........         46 Note
PLAT789_ALERT_4_G Atoms with Negative _atom_site_disorder_group  #         5 Check
PLAT790_ALERT_4_G Centre of Gravity not Within Unit Cell: Resd.  #        12 Note
              C2 H3 N
PLAT802_ALERT_4_G CIF Input Record(s) with more than 80 Characters         6 Info
PLAT811_ALERT_5_G No ADDSYM Analysis: Too Many Excluded Atoms ....         ! Info
PLAT860_ALERT_3_G Number of Least-Squares Restraints ............       1480 Note
PLAT868_ALERT_4_G ALERTS Due to the Use of _smtbx_masks Suppressed         ! Info
PLAT909_ALERT_3_G Percentage of I>2sig(I) Data at Theta(Max) Still       39% Note
PLAT978_ALERT_2_G Number C-C Bonds with Positive Residual Density.         0 Info

   1 ALERT level A = Most likely a serious problem - resolve or explain
   4 ALERT level B = A potentially serious problem, consider carefully
  26 ALERT level C = Check. Ensure it is not caused by an omission or oversight
 173 ALERT level G = General information/check it is not something unexpected

   5 ALERT type 1 CIF construction/syntax error, inconsistent or missing data
  39 ALERT type 2 Indicator that the structure model may be wrong or deficient
 112 ALERT type 3 Indicator that the structure quality may be low
  47 ALERT type 4 Improvement, methodology, query or suggestion
   1 ALERT type 5 Informative message, check
```

It is advisable to attempt to resolve as many as possible of the alerts in all categories. Often the minor alerts point to easily fixed oversights, errors and omissions in your CIF or refinement strategy, so attention to these fine details can be worthwhile. In order to resolve some of the more serious problems it may be necessary to carry out additional measurements or structure refinements. However, the purpose of your study may justify the reported deviations and the more serious of these should normally be commented upon in the discussion or experimental section of a paper or in the "special_details" fields of the CIF. checkCIF was carefully designed to identify outliers and unusual parameters, but every test has its limitations and alerts that are not important in a particular case may appear. Conversely, the absence of alerts does not guarantee there are no aspects of the results needing attention. It is up to the individual to critically assess their own results and, if necessary, seek expert advice.

Publication of your CIF in IUCr journals

A basic structural check has been run on your CIF. These basic checks will be run on all CIFs submitted for publication in IUCr journals (*Acta Crystallographica*, *Journal of Applied Crystallography*, *Journal of Synchrotron Radiation*); however, if you intend to submit to *Acta Crystallographica Section C* or *E* or *IUCrData*, you should make sure that full publication checks are run on the final version of your CIF prior to submission.

Publication of your CIF in other journals

Please refer to the *Notes for Authors* of the relevant journal for any special instructions relating to CIF submission.

PLATON version of 28/11/2022; check.def file version of 28/11/2022

Datablock mn - ellipsoid plot

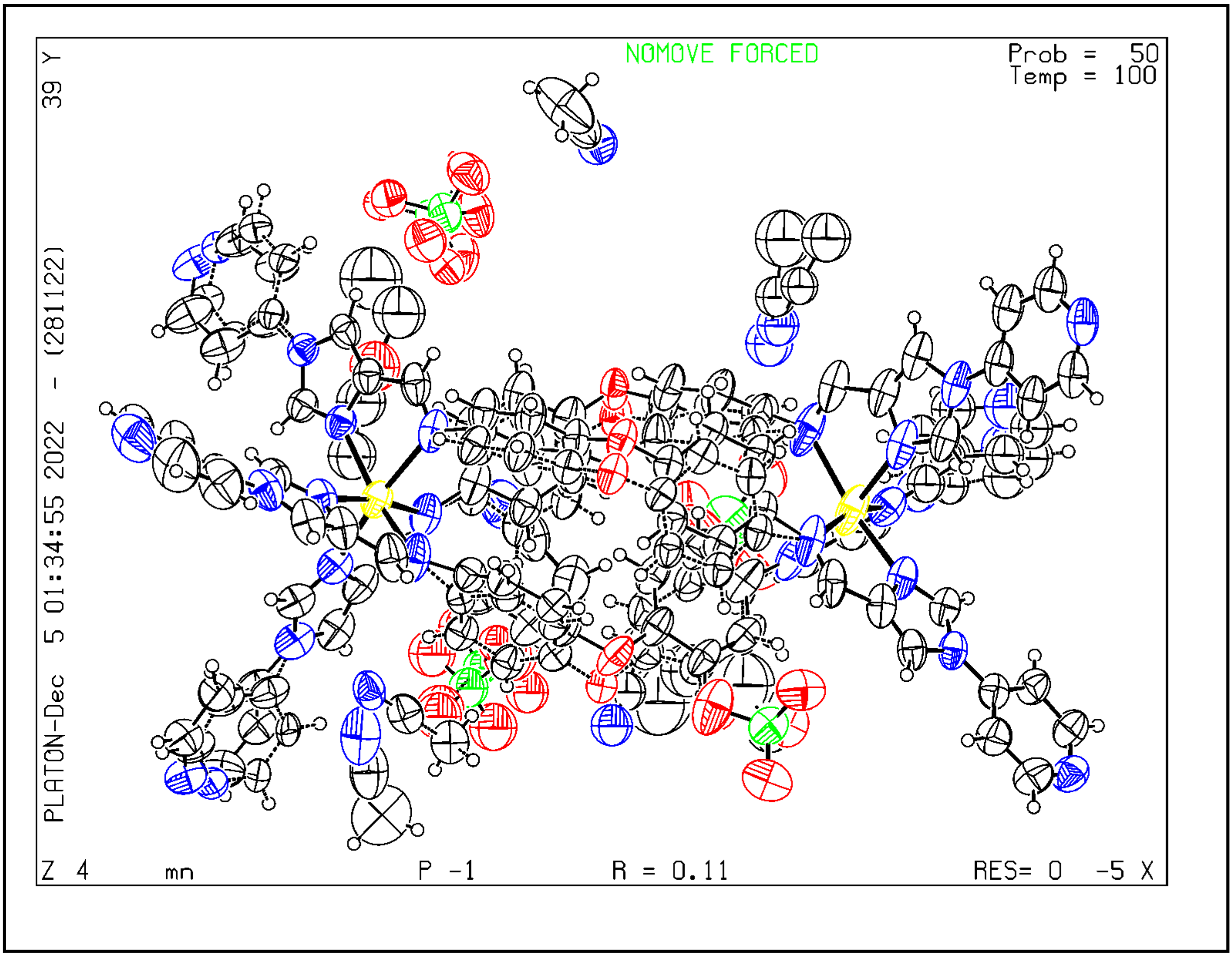

[Download CIF editor (publCIF) from the IUCr]
[Download CIF editor (enCIFer) from the CCDC]
[Test a new CIF entry]
