# Supplementary material for: Investigating the Conformations of a Family of [M2L3]4+ Helicates Using Single Crystal X-ray Diffraction
Source: Molecules. 2023 Feb 1;28(3):1404. doi: 10.3390/molecules28031404 (PMC9921459; doi:10.3390/molecules28031404)
Supplement: Supplementary file 1 [file molecules-28-01404-s001.zip › Final cifs/2224286.pdf]

No syntax errors found.
Please wait while processing ....

CIF dictionary
Interpreting this report

Datablock: cut83_a

Bond precision:	C-C = 0.0075 A		Wavelength=0.71073
Cell:	a=16.060(3)	b=17.710(4)	c=20.570(4)
	alpha=80.35(3)	beta=89.76(3)	gamma=72.80(3)
Temperature:	100 K		

	Calculated	Reported
Volume	5503(2)	5503(2)
Space group	P -1	P -1
Hall group	-P 1	-P 1
Moiety formula	2(C90 H66 Co2 N24 O3), 8(B F4), 2.667(C4 O), 6(C2 H3 N), C2 N [	C90 H66 Co2 N24 O3, 4(B F4), 3(C2 H3 N), 1.333(C4 O), 0.5(C2 N)
Sum formula	C204.67 H150 B8 Co4 F32 N55 O8.67 [+ solvent]	C102.34 H75 B4 Co2 F16 N27.50 O4.34
Mr	4448.66	2224.37
Dx,g cm-3	1.342	1.342
Z	1	2
Mu (mm-1)	0.392	0.392
F000	2268.3	2268.0
F000'	2270.84	
h,k,lmax	19,21,24	19,21,24
Nref	20165	19708
Tmin,Tmax	0.910,0.992	
Tmin'	0.889	

Correction method= Not given

Data completeness= 0.977 Theta(max)= 25.350

R(reflections)= 0.0914(15394) wR2(reflections)= 0.2925(19708)

S = 1.044 Npar= 1727

The following ALERTS were generated. Each ALERT has the format
test-name_ALERT_alert-type_alert-level.
Click on the hyperlinks for more details of the test.

Alert level A

PLAT201_ALERT_2_A Isotropic non-H Atoms in Main Residue(s) .... 11 Report
N1C N2C C1C C2C C3C etc.

Author Response: Orientational disorder was present in the ligand C terminal at the Co1 of the helicate molecule. Low electron density prohibited modelling a second po but may still affect anisotropic displacements, so the isotropic modelling was u

Alert level C

PLAT077_ALERT_4_C Unitcell Contains Non-integer Number of Atoms .. Please Check
PLAT084_ALERT_3_C High wR2 Value (i.e. > 0.25) .... 0.29 Report
PLAT202_ALERT_3_C Isotropic non-H Atoms in Anion/Solvent .... 8 Check
O1ET C1ET C2ET C3ET C4ET N1S4 etc.
PLAT234_ALERT_4_C Large Hirshfeld Difference C19A --C20A . 0.16 Ang.
PLAT241_ALERT_2_C High 'MainMol' Ueq as Compared to Neighbors of C21B Check
PLAT242_ALERT_2_C Low 'MainMol' Ueq as Compared to Neighbors of C16B Check
PLAT244_ALERT_4_C Low 'Solvent' Ueq as Compared to Neighbors of C1S1 Check
PLAT260_ALERT_2_C Large Average Ueq of Residue Including F1A 0.129 Check
And 6 other PLAT260 Alerts
More ...
PLAT334_ALERT_2_C Small <C-C> Benzene Dist. C10A -C15A . 1.36 Ang.
PLAT341_ALERT_3_C Low Bond Precision on C-C Bonds .... 0.00749 Ang.
PLAT767_ALERT_4_C INS Embedded LIST 6 Instruction Should be LIST 4 Please Check
PLAT906_ALERT_3_C Large K Value in the Analysis of Variance ...... 3.264 Check
PLAT911_ALERT_3_C Missing FCF Refl Between Thmin & STh/L= 0.600 398 Report
PLAT918_ALERT_3_C Reflection(s) with I(obs) much Smaller I(calc) . 4 Check
PLAT922_ALERT_1_C wR2 in the CIF and FCF Differ by .... -0.0021 Check
PLAT975_ALERT_2_C Check Calcd Resid. Dens. 1.07Ang From C3ET . 0.58 eA-3

Alert level G

PLAT002_ALERT_2_G Number of Distance or Angle Restraints on AtSite 133 Note
PLAT041_ALERT_1_G Calc. and Reported SumFormula Strings Differ Please Check
PLAT042_ALERT_1_G Calc. and Reported MoietyFormula Strings Differ Please Check

```
PLAT045_ALERT_1_G Calculated and Reported Z Differ by a Factor ...      0.500 Check
PLAT072_ALERT_2_G SHELXL First  Parameter in WGHT  Unusually Large       0.19 Report
PLAT154_ALERT_1_G The s.u.'s on the Cell Angles are Equal ..(Note)       0.03 Degree
PLAT172_ALERT_4_G The CIF-Embedded .res File Contains DFIX Records          8 Report
PLAT173_ALERT_4_G The CIF-Embedded .res File Contains DANG Records          8 Report
PLAT174_ALERT_4_G The CIF-Embedded .res File Contains FLAT Records         10 Report
PLAT176_ALERT_4_G The CIF-Embedded .res File Contains SADI Records        148 Report
PLAT180_ALERT_4_G Check Cell Rounding: # of Values Ending with 0 =          4 Note
PLAT187_ALERT_4_G The CIF-Embedded .res File Contains RIGU Records         19 Report
PLAT191_ALERT_3_G A Non-default SADI Restraint Value has been used     0.0400 Report
And 74 other PLAT191 Alerts
More ...
PLAT244_ALERT_4_G Low    'Solvent' Ueq as Compared to Neighbors of         B2 Check
PLAT300_ALERT_4_G Atom Site Occupancy of O4         Constrained at     0.3333 Check
And 7 other PLAT300 Alerts
More ...
PLAT301_ALERT_3_G Main Residue  Disorder .............(Resd  1  )        27% Note
PLAT302_ALERT_4_G Anion/Solvent/Minor-Residue Disorder (Resd  2  )       100% Note
And 13 other PLAT302 Alerts
More ...
PLAT304_ALERT_4_G Non-Integer Number of Atoms in ..... (Resd  2  )       2.75 Check
And 13 other PLAT304 Alerts
More ...
PLAT315_ALERT_2_G Singly Bonded Carbon Detected (H-atoms Missing).       C1ET Check
And 6 other PLAT315 Alerts
More ...
PLAT410_ALERT_2_G Short Intra H...H Contact  H7C      ..H4F      .       1.95 Ang.
                                                       x,y,z  =      1_555 Check
PLAT432_ALERT_2_G Short Inter X...Y Contact  F12B     ..C4B      .       2.91 Ang.
                                                     1+x,y,z  =      1_655 Check
PLAT432_ALERT_2_G Short Inter X...Y Contact  O1F      ..C24C     .       3.00 Ang.
                                                  1-x,1-y,-z  =      2_665 Check
PLAT605_ALERT_4_G Largest Solvent Accessible VOID in the Structure        103 A**3
PLAT720_ALERT_4_G Number of Unusual/Non-Standard Labels ..........         43 Note
PLAT789_ALERT_4_G Atoms with Negative _atom_site_disorder_group  #          5 Check
PLAT790_ALERT_4_G Centre of Gravity not Within Unit Cell: Resd.  #          5 Note
              B F4
And 3 other PLAT790 Alerts
More ...
PLAT802_ALERT_4_G CIF Input Record(s) with more than 80 Characters          3 Info
PLAT811_ALERT_5_G No ADDSYM Analysis: Too Many Excluded Atoms ....          ! Info
PLAT860_ALERT_3_G Number of Least-Squares Restraints ............        1021 Note
PLAT868_ALERT_4_G ALERTS Due to the Use of _smtbx_masks Suppressed          ! Info
PLAT883_ALERT_1_G No Info/Value for _atom_sites_solution_primary .     Please Do !
PLAT912_ALERT_4_G Missing # of FCF Reflections Above STh/L=  0.600         59 Note
PLAT933_ALERT_2_G Number of HKL-OMIT Records in Embedded .res File          1 Note
PLAT978_ALERT_2_G Number C-C Bonds with Positive Residual Density.          3 Info
```

```
   1 ALERT level A = Most likely a serious problem - resolve or explain
   0 ALERT level B = A potentially serious problem, consider carefully
  22 ALERT level C = Check. Ensure it is not caused by an omission or oversight
 150 ALERT level G = General information/check it is not something unexpected

   6 ALERT type 1 CIF construction/syntax error, inconsistent or missing data
  26 ALERT type 2 Indicator that the structure model may be wrong or deficient
  83 ALERT type 3 Indicator that the structure quality may be low
  57 ALERT type 4 Improvement, methodology, query or suggestion
   1 ALERT type 5 Informative message, check
```

It is advisable to attempt to resolve as many as possible of the alerts in all categories. Often the minor alerts point to easily fixed oversights, errors and omissions in your CIF or refinement strategy, so attention to these fine details can be worthwhile. In order to resolve some of the more serious problems it may be necessary to carry out additional measurements or structure refinements. However, the purpose of your study may justify the reported deviations and the more serious of these should normally be commented upon in the discussion or experimental section of a paper or in the "special_details" fields of the CIF. checkCIF was carefully designed to identify outliers and unusual parameters, but every test has its limitations and alerts that are not important in a particular case may appear. Conversely, the absence of alerts does not guarantee there are no aspects of the results needing attention. It is up to the individual to critically assess their own results and, if necessary, seek expert advice.

Publication of your CIF in IUCr journals

A basic structural check has been run on your CIF. These basic checks will be run on all CIFs submitted for publication in IUCr journals (*Acta Crystallographica*, *Journal of Applied Crystallography*, *Journal of Synchrotron Radiation*); however, if you intend to submit to *Acta Crystallographica Section C* or *E* or *IUCrData*, you should make sure that full publication checks are run on the final version of your CIF prior to submission.

Publication of your CIF in other journals

Please refer to the *Notes for Authors* of the relevant journal for any special instructions relating to CIF submission.

PLATON version of 28/11/2022; check.def file version of 28/11/2022

Datablock cut83_a - ellipsoid plot

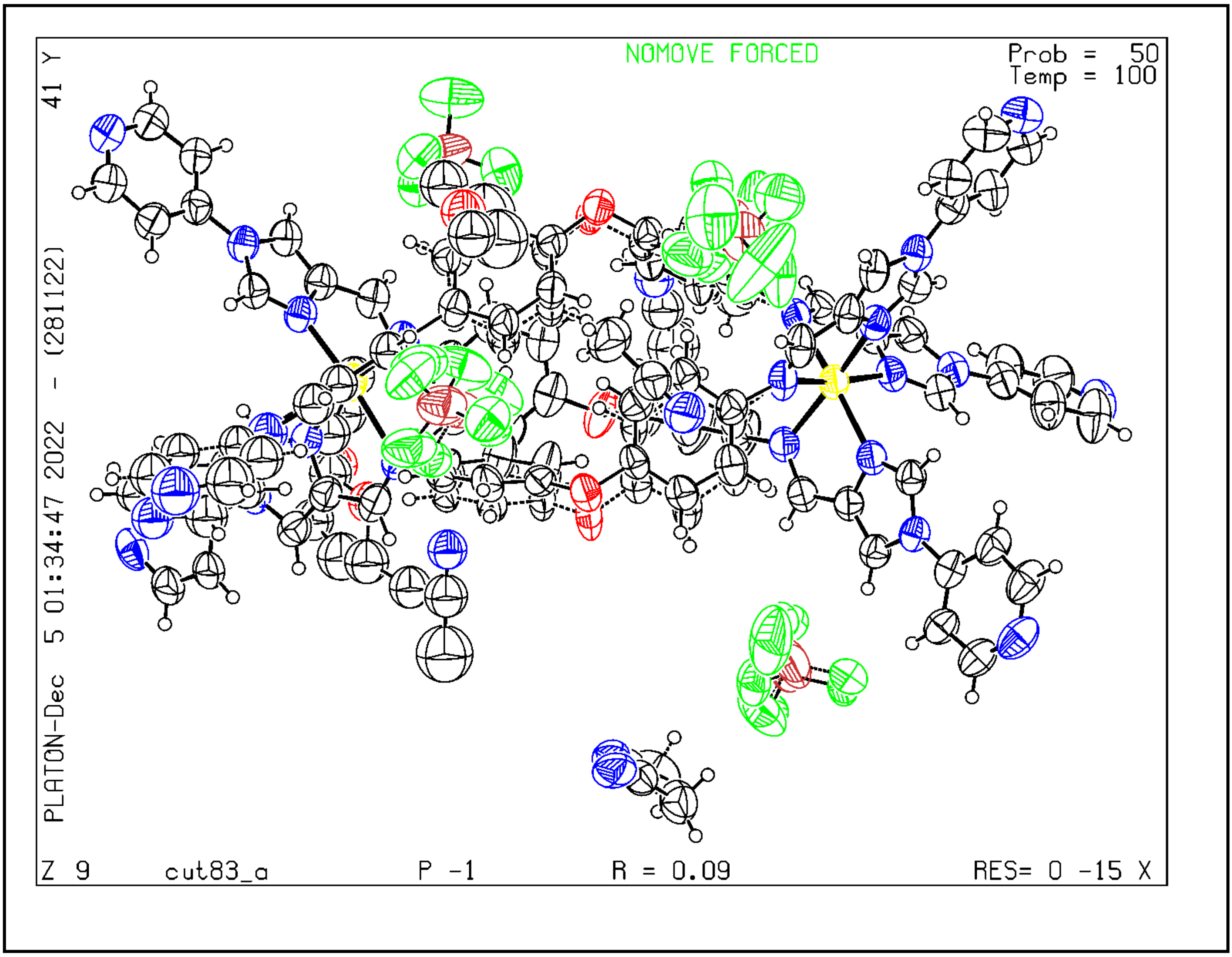

Download CIF editor (publCIF) from the IUCr
Download CIF editor (enCIFer) from the CCDC
Test a new CIF entry
