# Supplementary material for: Investigating the Conformations of a Family of [M2L3]4+ Helicates Using Single Crystal X-ray Diffraction
Source: Molecules. 2023 Feb 1;28(3):1404. doi: 10.3390/molecules28031404 (PMC9921459; doi:10.3390/molecules28031404)
Supplement: Supplementary file 1 [file molecules-28-01404-s001.zip › Final cifs/2224287.pdf]

No syntax errors found.
Please wait while processing ....

CIF dictionary
Interpreting this report

Datablock: cut81_a

Bond precision: C-C = 0.0085 A Wavelength=0.71073

Cell:	a=16.230(3)	b=17.690(4)	c=20.430(4)
	alpha=80.45(3)	beta=89.74(3)	gamma=72.24(3)

Temperature: 100 K

	Calculated	Reported
Volume	5502(2)	5502(2)
Space group	P -1	P -1
Hall group	-P 1	-P 1
Moiety formula	2(C90 H64 N24 Ni2 O3), 8(B F4), 2(C4 O), 3(C2 N), 4(C2 H3 N), 0	C90 H64 N24 Ni2 O3, 4(B F4), 1.5(C2 N), 2(C2 H3 N), C4 O, 0.25(
Sum formula	C202 H140 B8 F32 N55 Ni4 O8.50 [+ solvent]	C101 H70 B4 F16 N27.50 Ni2 O4.25
Mr	4402.94	2201.50
Dx,g cm-3	1.329	1.329
Z	1	2
Mu (mm-1)	0.432	0.432
F000	2245.0	2245.0
F000'	2247.46	
h,k,lmax	20,21,25	20,21,25
Nref	21676	20663
Tmin,Tmax	0.949,0.991	
Tmin'	0.917	

Correction method= Not given

Data completeness= 0.953 Theta(max)= 26.022

R(reflections)= 0.1109(16141) wR2(reflections)= 0.3899(20663)

S = 1.725 Npar= 1435

The following ALERTS were generated. Each ALERT has the format
test-name_ALERT_alert-type_alert-level.
Click on the hyperlinks for more details of the test.

Alert level A

PLAT201_ALERT_2_A Isotropic non-H Atoms in Main Residue(s) .... 19 Report
O1A N1C C1C C2C C3C etc.

Author Response: Weak high-angle reflections result in high Rint. This affects refineme parameters wR2, lowers bond precision and probably does not describe anisotropic displacements correctly. Orientational disorder in the helicate may give rise to some reflections which are not compatible with the assignment of Bravais lattice Orientational disorder in the linker segment of ligand A could not be satisfacto modelled with anisotropic displacement parameters.

PLAT934_ALERT_3_A Number of (Iobs-Icalc)/Sigma(W) > 10 Outliers .. 16 Check

Author Response: Weak high-angle reflections result in high Rint. This affects refineme parameters wR2, lowers bond precision and probably does not describe anisotropic displacements correctly. Orientational disorder in the helicate may give rise to some reflections which are not compatible with the assignment of Bravais lattice

Alert level B

PLAT084_ALERT_3_B High wR2 Value (i.e. > 0.25) .... 0.39 Report

Author Response: Weak high-angle reflections result in high Rint. This affects refineme parameters wR2, lowers bond precision and probably does not describe anisotropic displacements correctly. Orientational disorder in the helicate may give rise to some reflections which are not compatible with the assignment of Bravais lattice

PLAT315_ALERT_2_B Singly Bonded Carbon Detected (H-atoms Missing). C1ET Check

Author Response: Orientational disorder in some solvent molecules prevented anisotropic modelling, and in some cases hydrogen atoms were not fixed to allow the shift to converge to

zero.

```
PLAT315_ALERT_2_B Singly Bonded Carbon Detected (H-atoms Missing).       C4ET Check
```

Alert level C

```
PLAT029_ALERT_3_C _diffrn_measured_fraction_theta_full value Low .      0.979 Why?
PLAT077_ALERT_4_C Unitcell Contains Non-integer Number of Atoms ..     Please Check
PLAT082_ALERT_2_C High R1 Value ..................................       0.11 Report
PLAT202_ALERT_3_C Isotropic non-H Atoms in Anion/Solvent .........         16 Check
              F1A      F2A      F3A      F4A      B1A      O1ET            etc.
PLAT220_ALERT_2_C NonSolvent    Resd 1  C   Ueq(max)/Ueq(min) Range        3.2 Ratio
PLAT230_ALERT_2_C Hirshfeld Test Diff for     N4A      --C9A      .        6.2 s.u.
PLAT230_ALERT_2_C Hirshfeld Test Diff for     N8B      --C30B     .        5.3 s.u.
PLAT241_ALERT_2_C High   'MainMol' Ueq as Compared to Neighbors of         O1B Check
And 2 other PLAT241 Alerts
More ...
PLAT242_ALERT_2_C Low    'MainMol' Ueq as Compared to Neighbors of        C16B Check
PLAT243_ALERT_4_C High   'Solvent' Ueq as Compared to Neighbors of        O1ET Check
PLAT244_ALERT_4_C Low    'Solvent' Ueq as Compared to Neighbors of        C2ET Check
And 2 other PLAT244 Alerts
More ...
PLAT260_ALERT_2_C Large Average Ueq of Residue Including        F5      0.118 Check
And 4 other PLAT260 Alerts
More ...
PLAT334_ALERT_2_C Small <C-C> Benzene Dist.   C16A     -C21A     .       1.37 Ang.
PLAT334_ALERT_2_C Small <C-C> Benzene Dist.   C10D     -C15D     .       1.35 Ang.
PLAT341_ALERT_3_C Low Bond Precision on  C-C Bonds ...............    0.00847 Ang.
PLAT767_ALERT_4_C INS Embedded LIST 6 Instruction Should be LIST 4     Please Check
PLAT906_ALERT_3_C Large K Value in the Analysis of Variance ......      3.103 Check
PLAT911_ALERT_3_C Missing FCF Refl Between Thmin & STh/L=    0.600        427 Report
PLAT918_ALERT_3_C Reflection(s) with I(obs) much Smaller I(calc) .         16 Check
PLAT922_ALERT_1_C wR2 in the CIF and FCF Differ by ...............    -0.0011 Check
PLAT973_ALERT_2_C Check Calcd Positive Resid. Density on       Ni2       1.38 eA-3
PLAT973_ALERT_2_C Check Calcd Positive Resid. Density on       Ni1       1.32 eA-3
PLAT976_ALERT_2_C Check Calcd Resid. Dens.  0.76Ang From N1C     .      -0.45 eA-3
```

Alert level G

```
PLAT002_ALERT_2_G Number of Distance or Angle Restraints on AtSite        142 Note
PLAT003_ALERT_2_G Number of Uiso or Uij Restrained non-H Atoms ...          3 Report
PLAT042_ALERT_1_G Calc. and Reported MoietyFormula Strings  Differ     Please Check
PLAT045_ALERT_1_G Calculated and Reported Z Differ by a Factor ...      0.500 Check
PLAT072_ALERT_2_G SHELXL First  Parameter in WGHT  Unusually Large       0.20 Report
PLAT154_ALERT_1_G The s.u.'s on the Cell Angles are Equal ..(Note)       0.03 Degree
PLAT172_ALERT_4_G The CIF-Embedded .res File Contains DFIX Records         15 Report
PLAT173_ALERT_4_G The CIF-Embedded .res File Contains DANG Records          6 Report
PLAT174_ALERT_4_G The CIF-Embedded .res File Contains FLAT Records         13 Report
PLAT176_ALERT_4_G The CIF-Embedded .res File Contains SADI Records        120 Report
PLAT180_ALERT_4_G Check Cell Rounding: # of Values Ending with 0 =          3 Note
PLAT187_ALERT_4_G The CIF-Embedded .res File Contains RIGU Records         23 Report
PLAT191_ALERT_3_G A Non-default SADI Restraint Value has been used     0.0400 Report
And 57 other PLAT191 Alerts
More ...
PLAT244_ALERT_4_G Low    'Solvent' Ueq as Compared to Neighbors of          B2 Check
PLAT244_ALERT_4_G Low    'Solvent' Ueq as Compared to Neighbors of          B4 Check
PLAT300_ALERT_4_G Atom Site Occupancy of N1S3       Constrained at         0.5 Check
And 3 other PLAT300 Alerts
More ...
PLAT301_ALERT_3_G Main Residue  Disorder ..............(Resd  1  )         22% Note
PLAT302_ALERT_4_G Anion/Solvent/Minor-Residue Disorder (Resd  2  )        100% Note
And 9 other PLAT302 Alerts
More ...
PLAT304_ALERT_4_G Non-Integer Number of Atoms in ..... (Resd  2  )        2.54 Check
And 9 other PLAT304 Alerts
More ...
PLAT311_ALERT_2_G Isolated Disordered Oxygen Atom (No H's ?) .....         O1W Check
PLAT315_ALERT_2_G Singly Bonded Carbon Detected (H-atoms Missing).        C2S2 Check
```

Author Response: Orientational disorder in some solvent molecules prevented anisotropic modelling, and in some cases hydrogen atoms were not fixed to allow the shift to converge to zero.

```
And 2 other PLAT315 Alerts
More ...
PLAT343_ALERT_2_G Unusual sp?      Angle Range in Main Residue for         C9A Check
PLAT343_ALERT_2_G Unusual sp?      Angle Range in Main Residue for        C12B Check
PLAT344_ALERT_2_G Unusual sp?       Angle Range in Solvent/Ion for        C1ET Check
```

```
And 3 other PLAT344 Alerts
More ...
PLAT367_ALERT_2_G Long?  C(sp?)-C(sp?) Bond  C3ET     - C4ET     .       1.59 Ang.
PLAT398_ALERT_2_G Deviating  C-O-C   Angle From 120 for O1ET     .      137.9 Degree
PLAT432_ALERT_2_G Short Inter X...Y Contact  F12B      ..C4B     .       2.91 Ang.
                                                 -1+x,y,z  =     1_455 Check
PLAT432_ALERT_2_G Short Inter X...Y Contact  O1F       ..C24C    .       3.01 Ang.
                                               1-x,-y,1-z  =     2_656 Check
PLAT432_ALERT_2_G Short Inter X...Y Contact  C1C       ..C21D    .       3.15 Ang.
                                               1-x,1-y,-z  =     2_665 Check
PLAT432_ALERT_2_G Short Inter X...Y Contact  C3C       ..C12D    .       3.14 Ang.
                                               1-x,1-y,-z  =     2_665 Check
PLAT432_ALERT_2_G Short Inter X...Y Contact  C4C       ..C12D    .       2.69 Ang.
                                               1-x,1-y,-z  =     2_665 Check
PLAT432_ALERT_2_G Short Inter X...Y Contact  C4C       ..C11D    .       2.92 Ang.
                                               1-x,1-y,-z  =     2_665 Check
PLAT432_ALERT_2_G Short Inter X...Y Contact  C5C       ..C12D    .       2.83 Ang.
                                               1-x,1-y,-z  =     2_665 Check
PLAT432_ALERT_2_G Short Inter X...Y Contact  C5C       ..C11D    .       3.19 Ang.
                                               1-x,1-y,-z  =     2_665 Check
PLAT605_ALERT_4_G Largest Solvent Accessible VOID in the Structure        459 A**3
PLAT720_ALERT_4_G Number of Unusual/Non-Standard Labels ..........         32 Note
PLAT802_ALERT_4_G CIF Input Record(s) with more than 80 Characters          7 Info
PLAT811_ALERT_5_G No ADDSYM Analysis: Too Many Excluded Atoms ....          ! Info
PLAT860_ALERT_3_G Number of Least-Squares Restraints .............        912 Note
PLAT868_ALERT_4_G ALERTS Due to the Use of _smtbx_masks Suppressed          ! Info
PLAT883_ALERT_1_G No Info/Value for _atom_sites_solution_primary .     Please Do !
PLAT912_ALERT_4_G Missing # of FCF Reflections Above STh/L=  0.600        588 Note
PLAT978_ALERT_2_G Number C-C Bonds with Positive Residual Density.          0 Info
PLAT992_ALERT_5_G Repd & Actual _reflns_number_gt Values Differ by          4 Check
```

```
  2 ALERT level A = Most likely a serious problem - resolve or explain
  3 ALERT level B = A potentially serious problem, consider carefully
 31 ALERT level C = Check. Ensure it is not caused by an omission or oversight
127 ALERT level G = General information/check it is not something unexpected

  5 ALERT type 1 CIF construction/syntax error, inconsistent or missing data
 45 ALERT type 2 Indicator that the structure model may be wrong or deficient
 68 ALERT type 3 Indicator that the structure quality may be low
 43 ALERT type 4 Improvement, methodology, query or suggestion
  2 ALERT type 5 Informative message, check
```

It is advisable to attempt to resolve as many as possible of the alerts in all categories. Often the minor alerts point to easily fixed oversights, errors and omissions in your CIF or refinement strategy, so attention to these fine details can be worthwhile. In order to resolve some of the more serious problems it may be necessary to carry out additional measurements or structure refinements. However, the purpose of your study may justify the reported deviations and the more serious of these should normally be commented upon in the discussion or experimental section of a paper or in the "special_details" fields of the CIF. checkCIF was carefully designed to identify outliers and unusual parameters, but every test has its limitations and alerts that are not important in a particular case may appear. Conversely, the absence of alerts does not guarantee there are no aspects of the results needing attention. It is up to the individual to critically assess their own results and, if necessary, seek expert advice.

Publication of your CIF in IUCr journals

A basic structural check has been run on your CIF. These basic checks will be run on all CIFs submitted for publication in IUCr journals (*Acta Crystallographica*, *Journal of Applied Crystallography*, *Journal of Synchrotron Radiation*); however, if you intend to submit to *Acta Crystallographica Section C* or *E* or *IUCrData*, you should make sure that full publication checks are run on the final version of your CIF prior to submission.

Publication of your CIF in other journals

Please refer to the *Notes for Authors* of the relevant journal for any special instructions relating to CIF submission.

PLATON version of 28/11/2022; check.def file version of 28/11/2022

Datablock cut81_a - ellipsoid plot

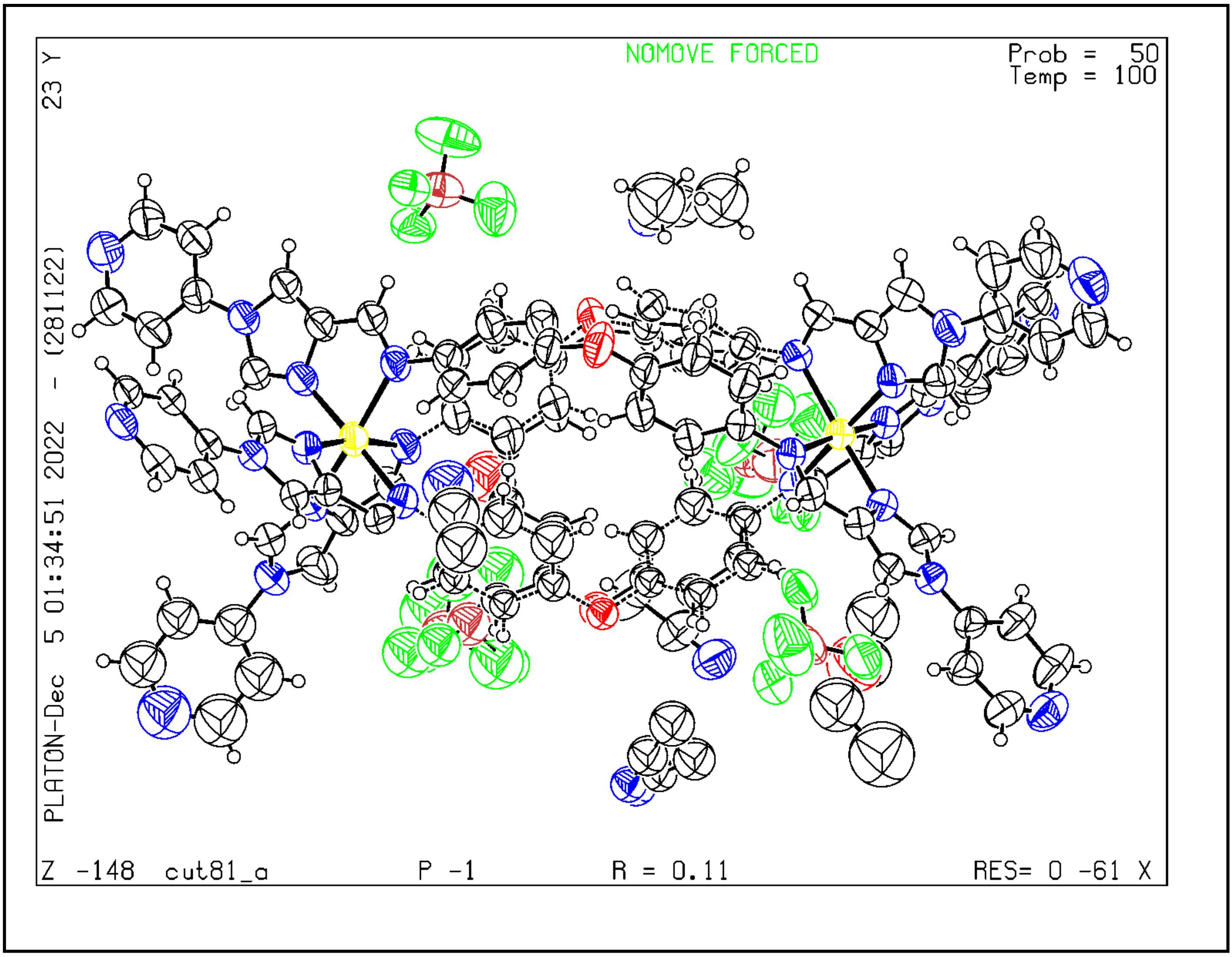

Download CIF editor (publCIF) from the IUCr
Download CIF editor (enCIFer) from the CCDC
Test a new CIF entry
