# Supplementary material for: Investigating the Conformations of a Family of [M2L3]4+ Helicates Using Single Crystal X-ray Diffraction
Source: Molecules. 2023 Feb 1;28(3):1404. doi: 10.3390/molecules28031404 (PMC9921459; doi:10.3390/molecules28031404)

checkCIF/PLATON report

Structure factors have been supplied for datablock(s) cut83_a

THIS REPORT IS FOR GUIDANCE ONLY. IF USED AS PART OF A REVIEW PROCEDURE FOR PUBLICATION, IT SHOULD NOT REPLACE THE EXPERTISE OF AN EXPERIENCED CRYSTALLOGRAPHIC REFEREE.

No syntax errors found. CIF dictionary Interpreting this report

Datablock: cut83_a

Bond precision: C-C = 0.0047 A Wavelength=0.71073

Cell:	a=19.950(4)	b=20.310(4)	c=40.550(8)
	alpha=91.68(3)	beta=99.57(3)	gamma=94.23(3)
Temperature:	100 K		

	Calculated	Reported
Volume	16143(6)	16143(6)
Space group	P -1	P -1
Hall group	-P 1	-P 1
Moiety formula	6(C90 H66 Cu2 N24 O3), 24(B F4), 4.667(C4 H10 O), C4 O, 33(C2 H	C90 H66 Cu2 N24 O3, 4(B F4), 5.5(C2 H3 N), 0.778(C4 H10 O), 0.1
Sum formula	C628.67 H541.67 B24 Cu12 F96 N177 O24.67	C104.78 H90.28 B4 Cu2 F16 N29.50 O4.11
Mr	13816.80	2302.78
Dx,g cm-3	1.421	1.421
Z	1	6
Mu (mm-1)	0.490	0.490
F000	7082.0	7082.0
F000'	7089.18	
h,k,lmax	24,25,50	24,24,50
Nref	63602	60876
Tmin,Tmax	0.889,0.952	
Tmin'	0.822	

Correction method= Not given

Data completeness= 0.957 Theta(max)= 26.022

```
R(reflections)= 0.0601( 51462)                              wR2(reflections)=
                                                            0.1838( 60876)
S = 1.021                   Npar= 4527
```

The following ALERTS were generated. Each ALERT has the format
test-name_ALERT_alert-type_alert-level.
Click on the hyperlinks for more details of the test.

Alert level B

```
PLAT201_ALERT_2_B Isotropic non-H Atoms in Main Residue(s) .......          6 Report
              N1F       C1F       C2F       C3F       C4F                  etc.
```

Author Response: Orientational disorder was present in one helicate (ligand F at the Cu3 end). The low electron density associated with the second part prevented its modelling, and this residual electron density caused problems in modelling the main part anisotropically.

Alert level C

```
PLAT077_ALERT_4_C Unitcell Contains Non-integer Number of Atoms ..     Please Check
PLAT202_ALERT_3_C Isotropic non-H Atoms in Anion/Solvent .........          8 Check
              O1ET    C2ET    C3ET    C1ET    C4ET    N1T2                 etc.
PLAT220_ALERT_2_C NonSolvent   Resd 2  C   Ueq(max)/Ueq(min) Range        3.3 Ratio
PLAT241_ALERT_2_C High   'MainMol' Ueq as Compared to Neighbors of        C5F Check
PLAT241_ALERT_2_C High   'MainMol' Ueq as Compared to Neighbors of       C12F Check
PLAT243_ALERT_4_C High   'Solvent' Ueq as Compared to Neighbors of       C3EU Check
PLAT244_ALERT_4_C Low    'Solvent' Ueq as Compared to Neighbors of       C1S1 Check
PLAT244_ALERT_4_C Low    'Solvent' Ueq as Compared to Neighbors of       C1T6 Check
PLAT244_ALERT_4_C Low    'Solvent' Ueq as Compared to Neighbors of       C1T1 Check
PLAT244_ALERT_4_C Low    'Solvent' Ueq as Compared to Neighbors of       C1S2 Check
PLAT244_ALERT_4_C Low    'Solvent' Ueq as Compared to Neighbors of       C1T9 Check
PLAT244_ALERT_4_C Low    'Solvent' Ueq as Compared to Neighbors of       C1T4 Check
PLAT244_ALERT_4_C Low    'Solvent' Ueq as Compared to Neighbors of       C1S3 Check
PLAT244_ALERT_4_C Low    'Solvent' Ueq as Compared to Neighbors of       C1S6 Check
PLAT244_ALERT_4_C Low    'Solvent' Ueq as Compared to Neighbors of       C1T2 Check
PLAT260_ALERT_2_C Large Average Ueq of Residue Including      O1EU      0.109 Check
PLAT260_ALERT_2_C Large Average Ueq of Residue Including      F17B      0.136 Check
PLAT260_ALERT_2_C Large Average Ueq of Residue Including      F45B      0.129 Check
PLAT260_ALERT_2_C Large Average Ueq of Residue Including      N1T9      0.114 Check
PLAT767_ALERT_4_C INS Embedded LIST 6 Instruction Should be LIST 4     Please Check
PLAT790_ALERT_4_C Centre of Gravity not Within Unit Cell: Resd.  #          1 Note
              C90 H66 Cu2 N24 O3
PLAT911_ALERT_3_C Missing FCF Refl Between Thmin & STh/L=    0.600       1090 Report
PLAT918_ALERT_3_C Reflection(s) with I(obs) much Smaller I(calc) .         22 Check
PLAT971_ALERT_2_C Check Calcd Resid. Dens.  0.71Ang From C1T8            1.80 eA-3
PLAT975_ALERT_2_C Check Calcd Resid. Dens.  1.04Ang From N1T7     .      1.45 eA-3
PLAT975_ALERT_2_C Check Calcd Resid. Dens.  0.94Ang From N1T8     .      0.84 eA-3
PLAT977_ALERT_2_C Check Negative Difference Density on H4F       .      -0.43 eA-3
```

Alert level G

```
FORMU01_ALERT_1_G  There is a discrepancy between the atom counts in the
            _chemical_formula_sum and _chemical_formula_moiety. This is
            usually due to the moiety formula being in the wrong format.
            Atom count from _chemical_formula_sum:   C104.7799 H90.28 B4 Cu2 F16 N
            Atom count from _chemical_formula_moiety:C104.7799 H90.28 B4 Cu2 F16 N
PLAT002_ALERT_2_G Number of Distance or Angle Restraints on AtSite         80 Note
PLAT045_ALERT_1_G Calculated and Reported Z Differ by a Factor ...      0.167 Check
PLAT072_ALERT_2_G SHELXL First  Parameter in WGHT  Unusually Large       0.11 Report
PLAT083_ALERT_2_G SHELXL Second Parameter in WGHT  Unusually Large      16.64 Why ?
PLAT154_ALERT_1_G The s.u.'s on the Cell Angles are Equal ..(Note)       0.03 Degree
PLAT171_ALERT_4_G The CIF-Embedded .res File Contains EADP Records          3 Report
PLAT172_ALERT_4_G The CIF-Embedded .res File Contains DFIX Records         12 Report
PLAT173_ALERT_4_G The CIF-Embedded .res File Contains DANG Records         12 Report
PLAT174_ALERT_4_G The CIF-Embedded .res File Contains FLAT Records          1 Report
PLAT176_ALERT_4_G The CIF-Embedded .res File Contains SADI Records         68 Report
PLAT180_ALERT_4_G Check Cell Rounding: # of Values Ending with 0 =          3 Note
PLAT187_ALERT_4_G The CIF-Embedded .res File Contains RIGU Records         15 Report
PLAT191_ALERT_3_G A Non-default SADI Restraint Value has been used     0.0400 Report
PLAT191_ALERT_3_G A Non-default SADI Restraint Value has been used     0.0400 Report
PLAT191_ALERT_3_G A Non-default SADI Restraint Value has been used     0.0400 Report
PLAT191_ALERT_3_G A Non-default SADI Restraint Value has been used     0.0400 Report
PLAT191_ALERT_3_G A Non-default SADI Restraint Value has been used     0.0400 Report
PLAT191_ALERT_3_G A Non-default SADI Restraint Value has been used     0.0400 Report
PLAT191_ALERT_3_G A Non-default SADI Restraint Value has been used     0.0400 Report
PLAT191_ALERT_3_G A Non-default SADI Restraint Value has been used     0.0400 Report
PLAT191_ALERT_3_G A Non-default SADI Restraint Value has been used     0.0400 Report
PLAT191_ALERT_3_G A Non-default SADI Restraint Value has been used     0.0400 Report
PLAT191_ALERT_3_G A Non-default SADI Restraint Value has been used     0.0400 Report
PLAT191_ALERT_3_G A Non-default SADI Restraint Value has been used     0.0400 Report
PLAT191_ALERT_3_G A Non-default SADI Restraint Value has been used     0.0400 Report
PLAT191_ALERT_3_G A Non-default SADI Restraint Value has been used     0.0400 Report
PLAT191_ALERT_3_G A Non-default SADI Restraint Value has been used     0.0400 Report
PLAT191_ALERT_3_G A Non-default SADI Restraint Value has been used     0.0400 Report
PLAT191_ALERT_3_G A Non-default SADI Restraint Value has been used     0.0400 Report
PLAT191_ALERT_3_G A Non-default SADI Restraint Value has been used     0.0400 Report
PLAT191_ALERT_3_G A Non-default SADI Restraint Value has been used     0.0400 Report
PLAT191_ALERT_3_G A Non-default SADI Restraint Value has been used     0.0400 Report
PLAT191_ALERT_3_G A Non-default SADI Restraint Value has been used     0.0400 Report
PLAT191_ALERT_3_G A Non-default SADI Restraint Value has been used     0.0400 Report
PLAT191_ALERT_3_G A Non-default SADI Restraint Value has been used     0.0400 Report
PLAT191_ALERT_3_G A Non-default SADI Restraint Value has been used     0.0400 Report
PLAT191_ALERT_3_G A Non-default SADI Restraint Value has been used     0.0400 Report
PLAT191_ALERT_3_G A Non-default SADI Restraint Value has been used     0.0400 Report
PLAT191_ALERT_3_G A Non-default SADI Restraint Value has been used     0.0400 Report
PLAT191_ALERT_3_G A Non-default SADI Restraint Value has been used     0.0400 Report
PLAT191_ALERT_3_G A Non-default SADI Restraint Value has been used     0.0400 Report
PLAT191_ALERT_3_G A Non-default SADI Restraint Value has been used     0.0400 Report
PLAT191_ALERT_3_G A Non-default SADI Restraint Value has been used     0.0400 Report
PLAT191_ALERT_3_G A Non-default SADI Restraint Value has been used     0.0400 Report
PLAT191_ALERT_3_G A Non-default SADI Restraint Value has been used     0.0400 Report
PLAT191_ALERT_3_G A Non-default SADI Restraint Value has been used     0.0400 Report
PLAT191_ALERT_3_G A Non-default SADI Restraint Value has been used     0.0400 Report
PLAT191_ALERT_3_G A Non-default SADI Restraint Value has been used     0.0400 Report
PLAT191_ALERT_3_G A Non-default SADI Restraint Value has been used     0.0400 Report
PLAT191_ALERT_3_G A Non-default SADI Restraint Value has been used     0.0400 Report
PLAT244_ALERT_4_G Low    'Solvent' Ueq as Compared to Neighbors of         B1 Check
PLAT244_ALERT_4_G Low    'Solvent' Ueq as Compared to Neighbors of         B2 Check
```

```
PLAT244_ALERT_4_G Low    'Solvent' Ueq as Compared to Neighbors of         B3 Check
PLAT244_ALERT_4_G Low    'Solvent' Ueq as Compared to Neighbors of         B6 Check
PLAT244_ALERT_4_G Low    'Solvent' Ueq as Compared to Neighbors of         B8 Check
PLAT244_ALERT_4_G Low    'Solvent' Ueq as Compared to Neighbors of         B9 Check
PLAT244_ALERT_4_G Low    'Solvent' Ueq as Compared to Neighbors of        B11 Check
PLAT300_ALERT_4_G Atom Site Occupancy of O1EW       Constrained at     0.6667 Check
PLAT300_ALERT_4_G Atom Site Occupancy of C4EW       Constrained at     0.6667 Check
PLAT300_ALERT_4_G Atom Site Occupancy of C2EW       Constrained at     0.6667 Check
PLAT300_ALERT_4_G Atom Site Occupancy of C1EW       Constrained at     0.6667 Check
PLAT300_ALERT_4_G Atom Site Occupancy of C3EW       Constrained at     0.6667 Check
PLAT300_ALERT_4_G Atom Site Occupancy of H4EA       Constrained at     0.6667 Check
PLAT300_ALERT_4_G Atom Site Occupancy of H4EB       Constrained at     0.6667 Check
PLAT300_ALERT_4_G Atom Site Occupancy of H4EC       Constrained at     0.6667 Check
PLAT300_ALERT_4_G Atom Site Occupancy of H2EA       Constrained at     0.6667 Check
PLAT300_ALERT_4_G Atom Site Occupancy of H2EB       Constrained at     0.6667 Check
PLAT300_ALERT_4_G Atom Site Occupancy of H1EA       Constrained at     0.6667 Check
PLAT300_ALERT_4_G Atom Site Occupancy of H1EB       Constrained at     0.6667 Check
PLAT300_ALERT_4_G Atom Site Occupancy of H1EC       Constrained at     0.6667 Check
PLAT300_ALERT_4_G Atom Site Occupancy of H3EA       Constrained at     0.6667 Check
PLAT300_ALERT_4_G Atom Site Occupancy of H3EB       Constrained at     0.6667 Check
PLAT300_ALERT_4_G Atom Site Occupancy of O1ET       Constrained at     0.6667 Check
PLAT300_ALERT_4_G Atom Site Occupancy of C2ET       Constrained at     0.6667 Check
PLAT300_ALERT_4_G Atom Site Occupancy of C3ET       Constrained at     0.6667 Check
PLAT300_ALERT_4_G Atom Site Occupancy of C1ET       Constrained at     0.6667 Check
PLAT300_ALERT_4_G Atom Site Occupancy of C4ET       Constrained at     0.6667 Check
PLAT300_ALERT_4_G Atom Site Occupancy of H2EE       Constrained at     0.6667 Check
PLAT300_ALERT_4_G Atom Site Occupancy of H2EF       Constrained at     0.6667 Check
PLAT300_ALERT_4_G Atom Site Occupancy of H3EE       Constrained at     0.6667 Check
PLAT300_ALERT_4_G Atom Site Occupancy of H3EF       Constrained at     0.6667 Check
PLAT300_ALERT_4_G Atom Site Occupancy of H1EG       Constrained at     0.6667 Check
PLAT300_ALERT_4_G Atom Site Occupancy of H1EH       Constrained at     0.6667 Check
PLAT300_ALERT_4_G Atom Site Occupancy of H1EI       Constrained at     0.6667 Check
PLAT300_ALERT_4_G Atom Site Occupancy of H4EG       Constrained at     0.6667 Check
PLAT300_ALERT_4_G Atom Site Occupancy of H4EH       Constrained at     0.6667 Check
PLAT300_ALERT_4_G Atom Site Occupancy of H4EI       Constrained at     0.6667 Check
PLAT300_ALERT_4_G Atom Site Occupancy of O1EV       Constrained at        0.5 Check
PLAT300_ALERT_4_G Atom Site Occupancy of C1EV       Constrained at        0.5 Check
PLAT300_ALERT_4_G Atom Site Occupancy of C2EV       Constrained at        0.5 Check
PLAT300_ALERT_4_G Atom Site Occupancy of C3EV       Constrained at        0.5 Check
PLAT300_ALERT_4_G Atom Site Occupancy of C4EV       Constrained at        0.5 Check
PLAT300_ALERT_4_G Atom Site Occupancy of N1T8       Constrained at        0.5 Check
PLAT300_ALERT_4_G Atom Site Occupancy of C1T8       Constrained at        0.5 Check
PLAT300_ALERT_4_G Atom Site Occupancy of C2T8       Constrained at        0.5 Check
PLAT300_ALERT_4_G Atom Site Occupancy of H2TV       Constrained at        0.5 Check
PLAT300_ALERT_4_G Atom Site Occupancy of H2TW       Constrained at        0.5 Check
PLAT300_ALERT_4_G Atom Site Occupancy of H2TX       Constrained at        0.5 Check
PLAT300_ALERT_4_G Atom Site Occupancy of N1T3       Constrained at     0.3333 Check
PLAT300_ALERT_4_G Atom Site Occupancy of C2T3       Constrained at     0.3333 Check
PLAT300_ALERT_4_G Atom Site Occupancy of C1T3       Constrained at     0.3333 Check
PLAT300_ALERT_4_G Atom Site Occupancy of H2TM       Constrained at     0.3333 Check
PLAT300_ALERT_4_G Atom Site Occupancy of H2TN       Constrained at     0.3333 Check
PLAT300_ALERT_4_G Atom Site Occupancy of H2TO       Constrained at     0.3333 Check
PLAT300_ALERT_4_G Atom Site Occupancy of N1T5       Constrained at     0.3333 Check
PLAT300_ALERT_4_G Atom Site Occupancy of C1T5       Constrained at     0.3333 Check
PLAT300_ALERT_4_G Atom Site Occupancy of C2T5       Constrained at     0.3333 Check
PLAT300_ALERT_4_G Atom Site Occupancy of H2TP       Constrained at     0.3333 Check
PLAT300_ALERT_4_G Atom Site Occupancy of H2TQ       Constrained at     0.3333 Check
```

```
PLAT300_ALERT_4_G Atom Site Occupancy of H2TR       Constrained at     0.3333 Check
PLAT300_ALERT_4_G Atom Site Occupancy of N1T7       Constrained at     0.3333 Check
PLAT300_ALERT_4_G Atom Site Occupancy of C1T7       Constrained at     0.3333 Check
PLAT300_ALERT_4_G Atom Site Occupancy of C2T7       Constrained at     0.3333 Check
PLAT300_ALERT_4_G Atom Site Occupancy of H2TY       Constrained at     0.3333 Check
PLAT300_ALERT_4_G Atom Site Occupancy of Hb         Constrained at     0.3333 Check
PLAT300_ALERT_4_G Atom Site Occupancy of Hc         Constrained at     0.3333 Check
PLAT300_ALERT_4_G Atom Site Occupancy of O1W        Constrained at        0.5 Check
PLAT302_ALERT_4_G Anion/Solvent/Minor-Residue Disorder (Resd  7  )       100% Note
PLAT302_ALERT_4_G Anion/Solvent/Minor-Residue Disorder (Resd  8  )       100% Note
PLAT302_ALERT_4_G Anion/Solvent/Minor-Residue Disorder (Resd  9  )       100% Note
PLAT302_ALERT_4_G Anion/Solvent/Minor-Residue Disorder (Resd 11  )       100% Note
PLAT302_ALERT_4_G Anion/Solvent/Minor-Residue Disorder (Resd 15  )       100% Note
PLAT302_ALERT_4_G Anion/Solvent/Minor-Residue Disorder (Resd 16  )       100% Note
PLAT302_ALERT_4_G Anion/Solvent/Minor-Residue Disorder (Resd 18  )       100% Note
PLAT302_ALERT_4_G Anion/Solvent/Minor-Residue Disorder (Resd 19  )       100% Note
PLAT302_ALERT_4_G Anion/Solvent/Minor-Residue Disorder (Resd 20  )       100% Note
PLAT302_ALERT_4_G Anion/Solvent/Minor-Residue Disorder (Resd 21  )       100% Note
PLAT302_ALERT_4_G Anion/Solvent/Minor-Residue Disorder (Resd 22  )       100% Note
PLAT302_ALERT_4_G Anion/Solvent/Minor-Residue Disorder (Resd 23  )       100% Note
PLAT302_ALERT_4_G Anion/Solvent/Minor-Residue Disorder (Resd 24  )       100% Note
PLAT302_ALERT_4_G Anion/Solvent/Minor-Residue Disorder (Resd 40  )       100% Note
PLAT302_ALERT_4_G Anion/Solvent/Minor-Residue Disorder (Resd 41  )       100% Note
PLAT302_ALERT_4_G Anion/Solvent/Minor-Residue Disorder (Resd 42  )       100% Note
PLAT302_ALERT_4_G Anion/Solvent/Minor-Residue Disorder (Resd 43  )       100% Note
PLAT302_ALERT_4_G Anion/Solvent/Minor-Residue Disorder (Resd 44  )       100% Note
PLAT304_ALERT_4_G Non-Integer Number of Atoms in ..... (Resd  7  )       3.40 Check
PLAT304_ALERT_4_G Non-Integer Number of Atoms in ..... (Resd  8  )       2.55 Check
PLAT304_ALERT_4_G Non-Integer Number of Atoms in ..... (Resd 11  )       2.75 Check
PLAT304_ALERT_4_G Non-Integer Number of Atoms in ..... (Resd 15  )       3.31 Check
PLAT304_ALERT_4_G Non-Integer Number of Atoms in ..... (Resd 19  )       2.50 Check
PLAT304_ALERT_4_G Non-Integer Number of Atoms in ..... (Resd 20  )       1.60 Check
PLAT304_ALERT_4_G Non-Integer Number of Atoms in ..... (Resd 22  )       2.45 Check
PLAT304_ALERT_4_G Non-Integer Number of Atoms in ..... (Resd 23  )       2.25 Check
PLAT304_ALERT_4_G Non-Integer Number of Atoms in ..... (Resd 24  )       1.69 Check
PLAT304_ALERT_4_G Non-Integer Number of Atoms in ..... (Resd 44  )       0.50 Check
PLAT311_ALERT_2_G Isolated Disordered Oxygen Atom (No H's ?) .....        O1W Check
PLAT315_ALERT_2_G Singly Bonded Carbon Detected (H-atoms Missing).       C1EV Check
PLAT315_ALERT_2_G Singly Bonded Carbon Detected (H-atoms Missing).       C4EV Check
PLAT413_ALERT_2_G Short Inter XH3 .. XHn     H7A       ..Hc       .       2.11 Ang.
                                                     x,y,z  =      1_555 Check
PLAT432_ALERT_2_G Short Inter X...Y Contact  F14A      ..C24B     .       2.89 Ang.
                                                   1+x,y,z  =      1_655 Check
PLAT432_ALERT_2_G Short Inter X...Y Contact  F18A      ..C7F      .       2.96 Ang.
                                                     x,y,z  =      1_555 Check
PLAT432_ALERT_2_G Short Inter X...Y Contact  F19A      ..C2T8     .       2.91 Ang.
                                                   x,1+y,z  =      1_565 Check
PLAT432_ALERT_2_G Short Inter X...Y Contact  C27C      ..C2T3     .       3.19 Ang.
                                                     x,y,z  =      1_555 Check
PLAT432_ALERT_2_G Short Inter X...Y Contact  C28C      ..C2T3     .       3.17 Ang.
                                                     x,y,z  =      1_555 Check
PLAT720_ALERT_4_G Number of Unusual/Non-Standard Labels .........         157 Note
PLAT789_ALERT_4_G Atoms with Negative _atom_site_disorder_group  #          5 Check
PLAT790_ALERT_4_G Centre of Gravity not Within Unit Cell: Resd.  #          2 Note
              C90 H66 Cu2 N24 O3
PLAT790_ALERT_4_G Centre of Gravity not Within Unit Cell: Resd.  #          3 Note
              C90 H66 Cu2 N24 O3
```

```
PLAT790_ALERT_4_G Centre of Gravity not Within Unit Cell: Resd.  #          4 Note
              B F4
PLAT790_ALERT_4_G Centre of Gravity not Within Unit Cell: Resd.  #          5 Note
              B F4
PLAT790_ALERT_4_G Centre of Gravity not Within Unit Cell: Resd.  #          6 Note
              B F4
PLAT790_ALERT_4_G Centre of Gravity not Within Unit Cell: Resd.  #          7 Note
              B F4
PLAT790_ALERT_4_G Centre of Gravity not Within Unit Cell: Resd.  #          8 Note
              B F4
PLAT790_ALERT_4_G Centre of Gravity not Within Unit Cell: Resd.  #          9 Note
              B F4
PLAT790_ALERT_4_G Centre of Gravity not Within Unit Cell: Resd.  #         10 Note
              B F4
PLAT790_ALERT_4_G Centre of Gravity not Within Unit Cell: Resd.  #         11 Note
              B F4
PLAT790_ALERT_4_G Centre of Gravity not Within Unit Cell: Resd.  #         12 Note
              B F4
PLAT790_ALERT_4_G Centre of Gravity not Within Unit Cell: Resd.  #         13 Note
              B F4
PLAT790_ALERT_4_G Centre of Gravity not Within Unit Cell: Resd.  #         14 Note
              B F4
PLAT790_ALERT_4_G Centre of Gravity not Within Unit Cell: Resd.  #         15 Note
              B F4
PLAT790_ALERT_4_G Centre of Gravity not Within Unit Cell: Resd.  #         16 Note
              C4 H10 O
PLAT790_ALERT_4_G Centre of Gravity not Within Unit Cell: Resd.  #         17 Note
              C4 H10 O
PLAT790_ALERT_4_G Centre of Gravity not Within Unit Cell: Resd.  #         18 Note
              C4 H10 O
PLAT790_ALERT_4_G Centre of Gravity not Within Unit Cell: Resd.  #         19 Note
              C4 O
PLAT790_ALERT_4_G Centre of Gravity not Within Unit Cell: Resd.  #         20 Note
              B F4
PLAT790_ALERT_4_G Centre of Gravity not Within Unit Cell: Resd.  #         21 Note
              B F4
PLAT790_ALERT_4_G Centre of Gravity not Within Unit Cell: Resd.  #         22 Note
              B F4
PLAT790_ALERT_4_G Centre of Gravity not Within Unit Cell: Resd.  #         23 Note
              B F4
PLAT790_ALERT_4_G Centre of Gravity not Within Unit Cell: Resd.  #         24 Note
              B F4
PLAT790_ALERT_4_G Centre of Gravity not Within Unit Cell: Resd.  #         25 Note
              C2 H3 N
PLAT790_ALERT_4_G Centre of Gravity not Within Unit Cell: Resd.  #         26 Note
              C2 H3 N
PLAT790_ALERT_4_G Centre of Gravity not Within Unit Cell: Resd.  #         27 Note
              C2 H3 N
PLAT790_ALERT_4_G Centre of Gravity not Within Unit Cell: Resd.  #         28 Note
              C2 H3 N
PLAT790_ALERT_4_G Centre of Gravity not Within Unit Cell: Resd.  #         29 Note
              C2 H3 N
PLAT790_ALERT_4_G Centre of Gravity not Within Unit Cell: Resd.  #         30 Note
              C2 H3 N
PLAT790_ALERT_4_G Centre of Gravity not Within Unit Cell: Resd.  #         31 Note
              C2 H3 N
PLAT790_ALERT_4_G Centre of Gravity not Within Unit Cell: Resd.  #         32 Note
```

```
              C2 H3 N
PLAT790_ALERT_4_G Centre of Gravity not Within Unit Cell: Resd.  #         33 Note
              C2 H3 N
PLAT790_ALERT_4_G Centre of Gravity not Within Unit Cell: Resd.  #         34 Note
              C2 H3 N
PLAT790_ALERT_4_G Centre of Gravity not Within Unit Cell: Resd.  #         35 Note
              C2 H3 N
PLAT790_ALERT_4_G Centre of Gravity not Within Unit Cell: Resd.  #         36 Note
              C2 H3 N
PLAT790_ALERT_4_G Centre of Gravity not Within Unit Cell: Resd.  #         37 Note
              C2 H3 N
PLAT790_ALERT_4_G Centre of Gravity not Within Unit Cell: Resd.  #         38 Note
              C2 H3 N
PLAT790_ALERT_4_G Centre of Gravity not Within Unit Cell: Resd.  #         39 Note
              C2 H3 N
PLAT790_ALERT_4_G Centre of Gravity not Within Unit Cell: Resd.  #         40 Note
              C2 H3 N
PLAT790_ALERT_4_G Centre of Gravity not Within Unit Cell: Resd.  #         41 Note
              C2 H3 N
PLAT790_ALERT_4_G Centre of Gravity not Within Unit Cell: Resd.  #         42 Note
              C2 H3 N
PLAT790_ALERT_4_G Centre of Gravity not Within Unit Cell: Resd.  #         43 Note
              C2 H3 N
PLAT790_ALERT_4_G Centre of Gravity not Within Unit Cell: Resd.  #         44 Note
              O
PLAT794_ALERT_5_G Tentative Bond Valency for Cu1       (I)       .       1.16 Info
PLAT794_ALERT_5_G Tentative Bond Valency for Cu2       (I)       .       1.20 Info
PLAT794_ALERT_5_G Tentative Bond Valency for Cu3       (II)      .       2.13 Info
PLAT794_ALERT_5_G Tentative Bond Valency for Cu4       (I)       .       1.22 Info
PLAT794_ALERT_5_G Tentative Bond Valency for Cu5       (I)       .       1.16 Info
PLAT794_ALERT_5_G Tentative Bond Valency for Cu6       (I)       .       1.16 Info
PLAT860_ALERT_3_G Number of Least-Squares Restraints ............        774 Note
PLAT883_ALERT_1_G No Info/Value for _atom_sites_solution_primary .     Please Do !
PLAT910_ALERT_3_G Missing # of FCF Reflection(s) Below Theta(Min).          1 Note
PLAT912_ALERT_4_G Missing # of FCF Reflections Above STh/L=  0.600       1621 Note
PLAT913_ALERT_3_G Missing # of Very Strong Reflections in FCF ....          3 Note
PLAT933_ALERT_2_G Number of HKL-OMIT Records in Embedded .res File          9 Note
PLAT978_ALERT_2_G Number C-C Bonds with Positive Residual Density.          1 Info
```

---

0 **ALERT level A** = Most likely a serious problem - resolve or explain
1 **ALERT level B** = A potentially serious problem, consider carefully
27 **ALERT level C** = Check. Ensure it is not caused by an omission or oversight
213 **ALERT level G** = General information/check it is not something unexpected

4 ALERT type 1 CIF construction/syntax error, inconsistent or missing data
26 ALERT type 2 Indicator that the structure model may be wrong or deficient
44 ALERT type 3 Indicator that the structure quality may be low
161 ALERT type 4 Improvement, methodology, query or suggestion
6 ALERT type 5 Informative message, check

It is advisable to attempt to resolve as many as possible of the alerts in all categories. Often the minor alerts point to easily fixed oversights, errors and omissions in your CIF or refinement strategy, so attention to these fine details can be worthwhile. In order to resolve some of the more serious problems it may be necessary to carry out additional measurements or structure refinements. However, the purpose of your study may justify the reported deviations and the more serious of these should normally be commented upon in the discussion or experimental section of a paper or in the "special_details" fields of the CIF. checkCIF was carefully designed to identify outliers and unusual parameters, but every test has its limitations and alerts that are not important in a particular case may appear. Conversely, the absence of alerts does not guarantee there are no aspects of the results needing attention. It is up to the individual to critically assess their own results and, if necessary, seek expert advice.

Publication of your CIF in IUCr journals

A basic structural check has been run on your CIF. These basic checks will be run on all CIFs submitted for publication in IUCr journals (*Acta Crystallographica*, *Journal of Applied Crystallography*, *Journal of Synchrotron Radiation*); however, if you intend to submit to *Acta Crystallographica Section C* or *E* or *IUCrData*, you should make sure that full publication checks are run on the final version of your CIF prior to submission.

Publication of your CIF in other journals

Please refer to the *Notes for Authors* of the relevant journal for any special instructions relating to CIF submission.

PLATON version of 28/11/2022; check.def file version of 28/11/2022

Datablock cut83_a - ellipsoid plot

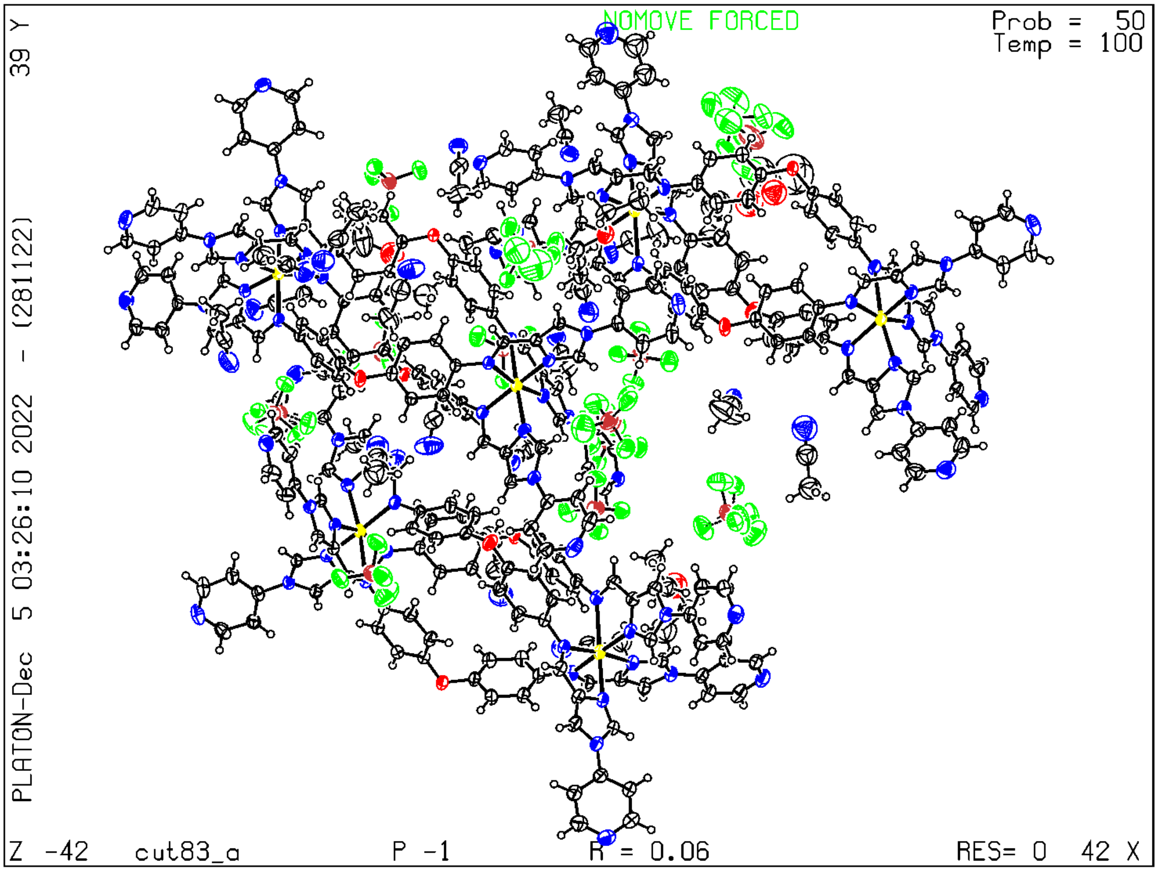

Supplement: Supplementary file 1 [file molecules-28-01404-s001.zip › Final cifs/2224288.pdf]
