# Supplementary material for: Investigating the Conformations of a Family of [M2L3]4+ Helicates Using Single Crystal X-ray Diffraction
Source: Molecules. 2023 Feb 1;28(3):1404. doi: 10.3390/molecules28031404 (PMC9921459; doi:10.3390/molecules28031404)
Supplement: Supplementary file 1 [file molecules-28-01404-s001.zip › Final cifs/2224289.pdf]

No syntax errors found.
Please wait while processing ....

CIF dictionary
Interpreting this report

Datablock: cut95_a

Bond precision:	C-C = 0.0130 A		Wavelength=0.71073
Cell:	a=16.120(3)	b=17.850(4)	c=20.560(4)
	alpha=80.07(3)	beta=89.62(3)	gamma=72.86(3)
Temperature:	100 K		

	Calculated	Reported
Volume	5562(2)	5562(2)
Space group	P -1	P -1
Hall group	-P 1	-P 1
Moiety formula	C90 H65 N24 O3 Zn2, 4(B F4), C4 H10 O, 3(C2 H3 N), C2 N, 0.25(O	C90 H65 N24 O3 Zn2, 4(B F4), 3(C2 H3 N), C4 H10 O, C2 N, 0.25(O
Sum formula	C102 H84 B4 F16 N28 O4.25 Zn2 [+ solvent]	C102 H84 B4 F16 N28 O4.25 Zn2
Mr	2247.99	2247.95
Dx,g cm-3	1.342	1.342
Z	2	2
Mu (mm-1)	0.521	0.521
F000	2300.0	2300.0
F000'	2302.24	
h,k,lmax	16,18,21	16,18,21
Nref	13576	13475
Tmin,Tmax	0.901,0.990	
Tmin'	0.901	

Correction method= Not given

Data completeness= 0.993 Theta(max)= 21.967

R(reflections)= 0.0998(9437) wR2(reflections)= 0.3389(13475)

S = 1.397 Npar= 1632

The following ALERTS were generated. Each ALERT has the format
test-name_ALERT_alert-type_alert-level.
Click on the hyperlinks for more details of the test.

Alert level A

THETM01_ALERT_3_A The value of sine(theta_max)/wavelength is less than 0.550
Calculated sin(theta_max)/wavelength = 0.5263

Author Response: The crystal did not diffract to high angles, so the theta_max value is

PLAT201_ALERT_2_A Isotropic non-H Atoms in Main Residue(s) .... 12 Report
N1C N2C N5C C1C C2C etc.

Author Response: Orientational disorder was present in the ligand C terminal at the Zn1 of the helicate molecule. Low electron density prohibited modelling a second po but may still affect anisotropic displacements, so the isotropic modelling was u

---

Alert level C

PLAT077_ALERT_4_C Unitcell Contains Non-integer Number of Atoms .. Please Check
PLAT084_ALERT_3_C High wR2 Value (i.e. > 0.25) .... 0.34 Report
PLAT088_ALERT_3_C Poor Data / Parameter Ratio .... 8.26 Note
PLAT202_ALERT_3_C Isotropic non-H Atoms in Anion/Solvent .... 8 Check
O1ET C1ET C2ET C3ET C4ET N1S3 etc.
PLAT234_ALERT_4_C Large Hirshfeld Difference N5A --C22A . 0.19 Ang.
And 3 other PLAT234 Alerts
More ...
PLAT241_ALERT_2_C High 'MainMol' Ueq as Compared to Neighbors of O1B Check
And 5 other PLAT241 Alerts
More ...
PLAT242_ALERT_2_C Low 'MainMol' Ueq as Compared to Neighbors of C16B Check
PLAT244_ALERT_4_C Low 'Solvent' Ueq as Compared to Neighbors of C1S1 Check
PLAT260_ALERT_2_C Large Average Ueq of Residue Including F1A 0.160 Check
And 10 other PLAT260 Alerts
More ...
PLAT334_ALERT_2_C Small <C-C> Benzene Dist. C10A -C15A . 1.36 Ang.
PLAT341_ALERT_3_C Low Bond Precision on C-C Bonds .... 0.01304 Ang.
PLAT360_ALERT_2_C Short C(sp3)-C(sp3) Bond C1ET - C2ET . 1.41 Ang.

```
PLAT767_ALERT_4_C INS Embedded LIST 6 Instruction Should be LIST 4     Please Check
PLAT911_ALERT_3_C Missing FCF Refl Between Thmin & STh/L=    0.526        101 Report
PLAT918_ALERT_3_C Reflection(s) with I(obs) much Smaller I(calc) .          2 Check
PLAT922_ALERT_1_C wR2 in the CIF and FCF Differ by ..............     -0.0039 Check
PLAT923_ALERT_1_C S    Values in the CIF and FCF Differ by .......     -0.017 Check
PLAT934_ALERT_3_C Number of (Iobs-Icalc)/Sigma(W) > 10 Outliers ..          1 Check
```

Alert level G

```
PLAT002_ALERT_2_G Number of Distance or Angle Restraints on AtSite        128 Note
PLAT003_ALERT_2_G Number of Uiso or Uij Restrained non-H Atoms ...          6 Report
PLAT072_ALERT_2_G SHELXL First  Parameter in WGHT  Unusually Large       0.20 Report
PLAT154_ALERT_1_G The s.u.'s on the Cell Angles are Equal ..(Note)       0.03 Degree
PLAT171_ALERT_4_G The CIF-Embedded .res File Contains EADP Records          6 Report
PLAT172_ALERT_4_G The CIF-Embedded .res File Contains DFIX Records          9 Report
PLAT173_ALERT_4_G The CIF-Embedded .res File Contains DANG Records          3 Report
PLAT174_ALERT_4_G The CIF-Embedded .res File Contains FLAT Records         12 Report
PLAT176_ALERT_4_G The CIF-Embedded .res File Contains SADI Records        121 Report
PLAT180_ALERT_4_G Check Cell Rounding: # of Values Ending with 0 =          3 Note
PLAT187_ALERT_4_G The CIF-Embedded .res File Contains RIGU Records         18 Report
PLAT191_ALERT_3_G A Non-default SADI Restraint Value has been used     0.0400 Report
```
And 59 other PLAT191 Alerts
More ...
```
PLAT244_ALERT_4_G Low    'Solvent' Ueq as Compared to Neighbors of         B2 Check
PLAT244_ALERT_4_G Low    'Solvent' Ueq as Compared to Neighbors of         B4 Check
PLAT300_ALERT_4_G Atom Site Occupancy of O1W        Constrained at        0.5 Check
PLAT301_ALERT_3_G Main Residue  Disorder .............(Resd  1  )         22% Note
PLAT302_ALERT_4_G Anion/Solvent/Minor-Residue Disorder (Resd  2  )       100% Note
```
And 10 other PLAT302 Alerts
More ...
```
PLAT304_ALERT_4_G Non-Integer Number of Atoms in ..... (Resd  2  )       3.25 Check
```
And 10 other PLAT304 Alerts
More ...
```
PLAT311_ALERT_2_G Isolated Disordered Oxygen Atom (No H's ?) .....        O1W Check
PLAT315_ALERT_2_G Singly Bonded Carbon Detected (H-atoms Missing).       C2S4 Check
PLAT315_ALERT_2_G Singly Bonded Carbon Detected (H-atoms Missing).       C2T4 Check
PLAT343_ALERT_2_G Unusual sp?      Angle Range in Main Residue for        C6B Check
PLAT411_ALERT_2_G Short Inter H...H Contact  H1C      ..H21D     .       2.00 Ang.
                                              1-x,2-y,-z  =      2_675 Check
PLAT413_ALERT_2_G Short Inter XH3 .. XHn     H4EB     ..H2TB     .       1.75 Ang.
                                                   x,y,z  =      1_555 Check
PLAT413_ALERT_2_G Short Inter XH3 .. XHn     H4EC     ..H2TC     .       1.87 Ang.
                                                   x,y,z  =      1_555 Check
PLAT432_ALERT_2_G Short Inter X...Y Contact  F12B     ..C4B      .       2.96 Ang.
                                                -1+x,y,z  =      1_455 Check
PLAT432_ALERT_2_G Short Inter X...Y Contact  O1F      ..C24C     .       3.02 Ang.
                                             1-x,1-y,1-z  =      2_666 Check
```
And 2 other PLAT432 Alerts
More ...
```
                                              1-x,2-y,-z  =      2_675 Check
PLAT432_ALERT_2_G Short Inter X...Y Contact  C1C      ..C21D     .       3.01 Ang.
                                              1-x,2-y,-z  =      2_675 Check
PLAT432_ALERT_2_G Short Inter X...Y Contact  C3C      ..C12D     .       3.02 Ang.
                                              1-x,2-y,-z  =      2_675 Check
PLAT432_ALERT_2_G Short Inter X...Y Contact  C3C      ..C13D     .       3.09 Ang.
                                              1-x,2-y,-z  =      2_675 Check
PLAT432_ALERT_2_G Short Inter X...Y Contact  C4C      ..C12D     .       2.54 Ang.
                                              1-x,2-y,-z  =      2_675 Check
PLAT432_ALERT_2_G Short Inter X...Y Contact  C4C      ..C11D     .       2.81 Ang.
                                              1-x,2-y,-z  =      2_675 Check
PLAT432_ALERT_2_G Short Inter X...Y Contact  C4C      ..C13D     .       3.19 Ang.
                                              1-x,2-y,-z  =      2_675 Check
PLAT432_ALERT_2_G Short Inter X...Y Contact  C5C      ..C12D     .       2.61 Ang.
                                              1-x,2-y,-z  =      2_675 Check
PLAT432_ALERT_2_G Short Inter X...Y Contact  C5C      ..C11D     .       3.03 Ang.
                                              1-x,2-y,-z  =      2_675 Check
```
And 3 other PLAT432 Alerts
More ...
```
                                             1-x,2-y,1-z  =      2_676 Check
PLAT605_ALERT_4_G Largest Solvent Accessible VOID in the Structure        463 A**3
PLAT720_ALERT_4_G Number of Unusual/Non-Standard Labels ..........         51 Note
PLAT722_ALERT_1_G Angle   Calc      121.00, Rep      119.90 Dev...       1.10 Degree
              C17D    -C18D    -H18D       1_555  1_555  1_555     #  468 Check
PLAT790_ALERT_4_G Centre of Gravity not Within Unit Cell: Resd.  #          5 Note
              B F4
```
And 2 other PLAT790 Alerts
More ...
```
PLAT802_ALERT_4_G CIF Input Record(s) with more than 80 Characters          4 Info
PLAT811_ALERT_5_G No ADDSYM Analysis: Too Many Excluded Atoms ....          ! Info
```

```
PLAT860_ALERT_3_G Number of Least-Squares Restraints .............       1093 Note
PLAT868_ALERT_4_G ALERTS Due to the Use of _smtbx_masks Suppressed          ! Info
PLAT883_ALERT_1_G No Info/Value for _atom_sites_solution_primary .     Please Do !
PLAT909_ALERT_3_G Percentage of I>2sig(I) Data at Theta(Max) Still        34% Note
PLAT978_ALERT_2_G Number C-C Bonds with Positive Residual Density.          0 Info
```

```
   2 ALERT level A = Most likely a serious problem - resolve or explain
   0 ALERT level B = A potentially serious problem, consider carefully
  36 ALERT level C = Check. Ensure it is not caused by an omission or oversight
 132 ALERT level G = General information/check it is not something unexpected

   5 ALERT type 1 CIF construction/syntax error, inconsistent or missing data
  47 ALERT type 2 Indicator that the structure model may be wrong or deficient
  71 ALERT type 3 Indicator that the structure quality may be low
  46 ALERT type 4 Improvement, methodology, query or suggestion
   1 ALERT type 5 Informative message, check
```

It is advisable to attempt to resolve as many as possible of the alerts in all categories. Often the minor alerts point to easily fixed oversights, errors and omissions in your CIF or refinement strategy, so attention to these fine details can be worthwhile. In order to resolve some of the more serious problems it may be necessary to carry out additional measurements or structure refinements. However, the purpose of your study may justify the reported deviations and the more serious of these should normally be commented upon in the discussion or experimental section of a paper or in the "special_details" fields of the CIF. checkCIF was carefully designed to identify outliers and unusual parameters, but every test has its limitations and alerts that are not important in a particular case may appear. Conversely, the absence of alerts does not guarantee there are no aspects of the results needing attention. It is up to the individual to critically assess their own results and, if necessary, seek expert advice.

Publication of your CIF in IUCr journals

A basic structural check has been run on your CIF. These basic checks will be run on all CIFs submitted for publication in IUCr journals (*Acta Crystallographica*, *Journal of Applied Crystallography*, *Journal of Synchrotron Radiation*); however, if you intend to submit to *Acta Crystallographica Section C* or *E* or *IUCrData*, you should make sure that full publication checks are run on the final version of your CIF prior to submission.

Publication of your CIF in other journals

Please refer to the *Notes for Authors* of the relevant journal for any special instructions relating to CIF submission.

PLATON version of 28/11/2022; check.def file version of 28/11/2022

Datablock cut95_a - ellipsoid plot

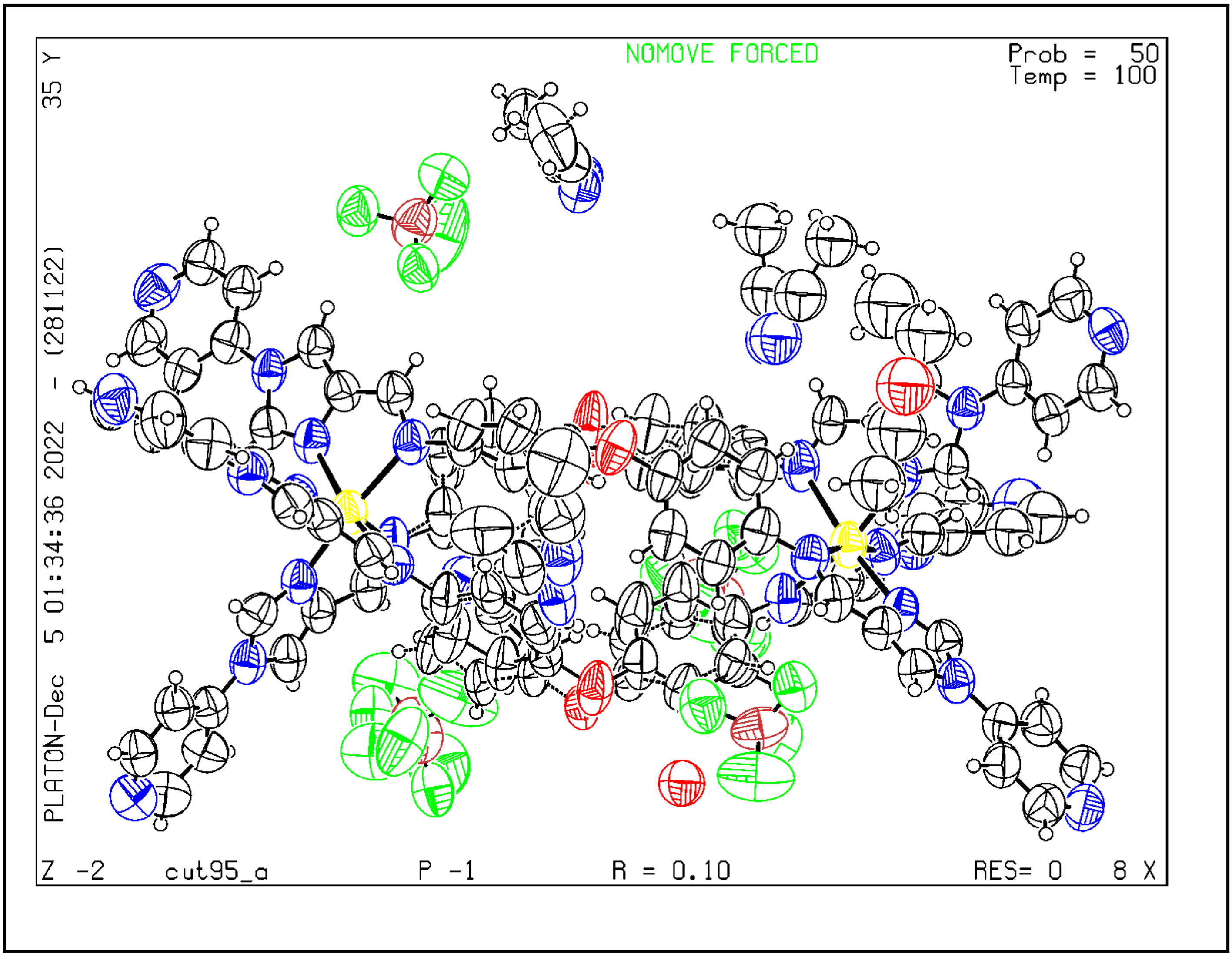

[Download CIF editor (publCIF) from the IUCr](#)
[Download CIF editor (enCIFer) from the CCDC](#)
[Test a new CIF entry](#)
