# Supplementary material for: Investigating the Conformations of a Family of [M2L3]4+ Helicates Using Single Crystal X-ray Diffraction
Source: Molecules. 2023 Feb 1;28(3):1404. doi: 10.3390/molecules28031404 (PMC9921459; doi:10.3390/molecules28031404)
Supplement: Supplementary file 1 [file molecules-28-01404-s001.zip › molecules-2189619-supplementary.pdf]

Supporting Information

Investigating the Conformations of a Family of $[M_2L_3]^{4+}$ Helicates Using Single Crystal X-Ray Diffraction

Matthew J. Wallis[1], Hyunsung Min [1], Leonard F. Lindoy [2] and Feng Li. [1*]

[1]School of Science, Western Sydney University, Locked Bag 1797, Penrith, Sydney, NSW 2751, Australia; 17232236@student.westernsydney.edu.au (M.J.W.); 17792947@student.westernsydney.edu.au (H.M.)
[2]School of Chemistry, University of Sydney, NSW 2006, Australia; leonard.lindoy@sydney.edu.au (L.F.L)
[*]Correspondence: Feng.Li@westernsydney.edu.au; Tel: +61-2-9685-9987

S1. HR ESI-MS and SEM-EDS for $[Mn_2\mathbf{L}_3](ClO_4)_4$**,** $[Co_2\mathbf{L}_3](BF_4)_4$**,** $[Ni_2\mathbf{L}_3](BF_4)_4$**,** $[Cu_2\mathbf{L}_3](BF_4)_4$ and $[Zn_2\mathbf{L}_3](BF_4)_4$.

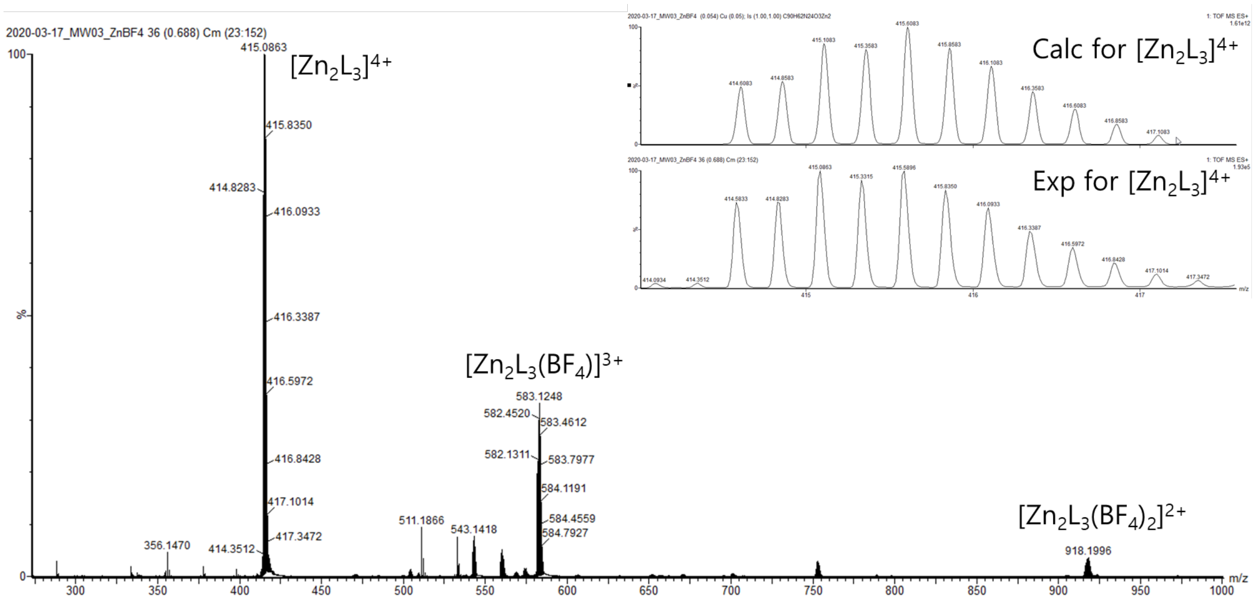

Figure S1. HR ESI-MS spectra for $[Zn_2\mathbf{L}_3](BF_4)_4$ in acetonitrile. Inset shows the isotopic pattern for $[Zn_2\mathbf{L}_3]^{4+}$ (bottom) with simulated pattern (top).

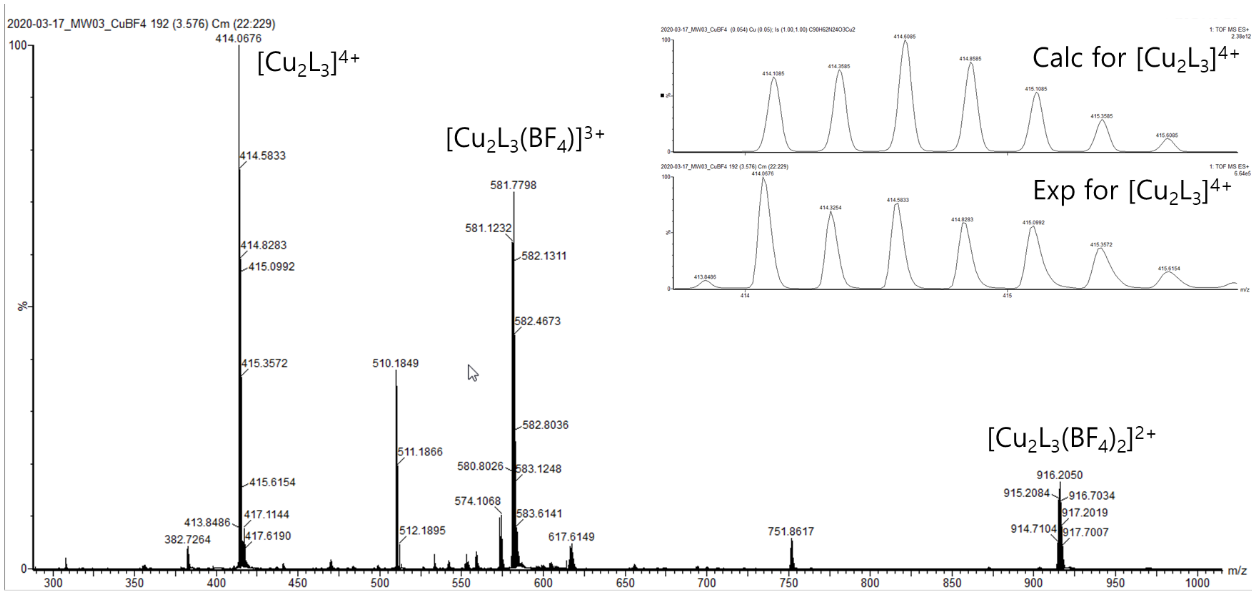

Figure S2. HR ESI-MS spectra for $[Cu_2\mathbf{L}_3](BF_4)_4$ in acetonitrile. Inset shows the isotopic pattern for $[Cu_2\mathbf{L}_3]^{4+}$ (bottom) with simulated pattern (top).

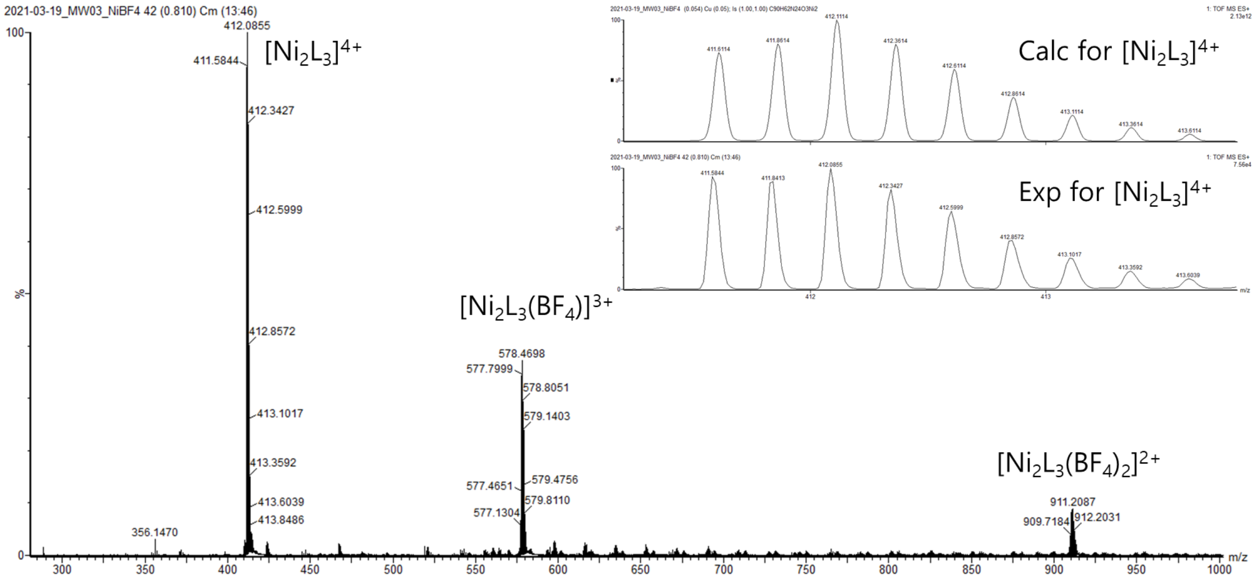

Figure S3. HR ESI-MS spectra for $[Ni_2\mathbf{L}_3](BF_4)_4$ in acetonitrile. Inset shows the isotopic pattern for $[Ni_2\mathbf{L}_3]^{4+}$ (bottom) with simulated pattern (top).

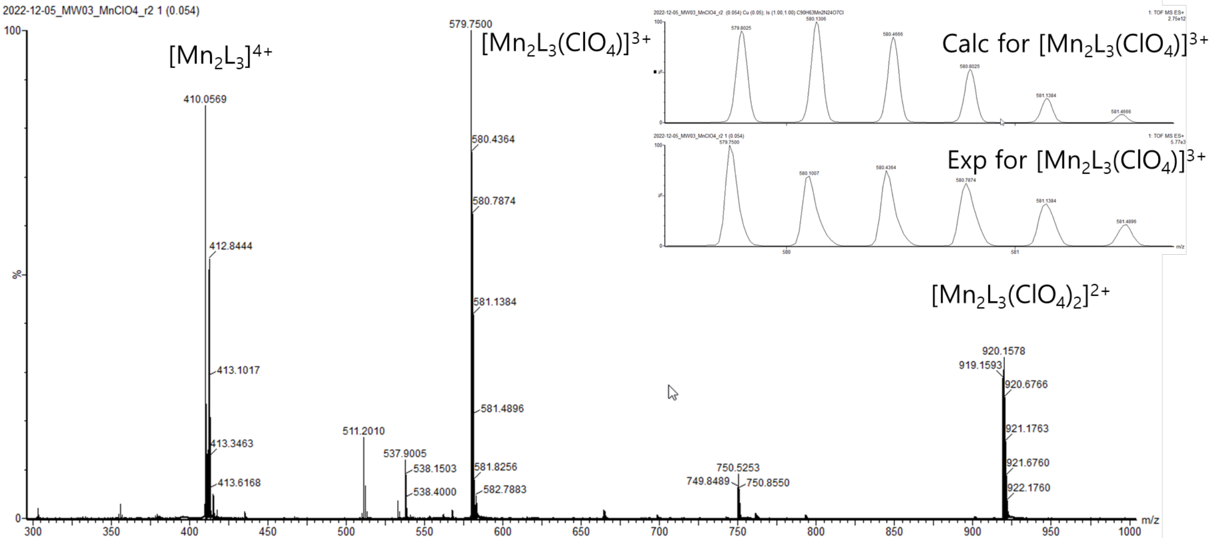

Figure S4. HR ESI-MS spectra for $[Mn_2\mathbf{L}_3](ClO_4)_4$ in acetonitrile. Inset shows the isotopic pattern for $[Mn_2L_3(ClO_4)]^{3+}$ (bottom) with simulated pattern (top).

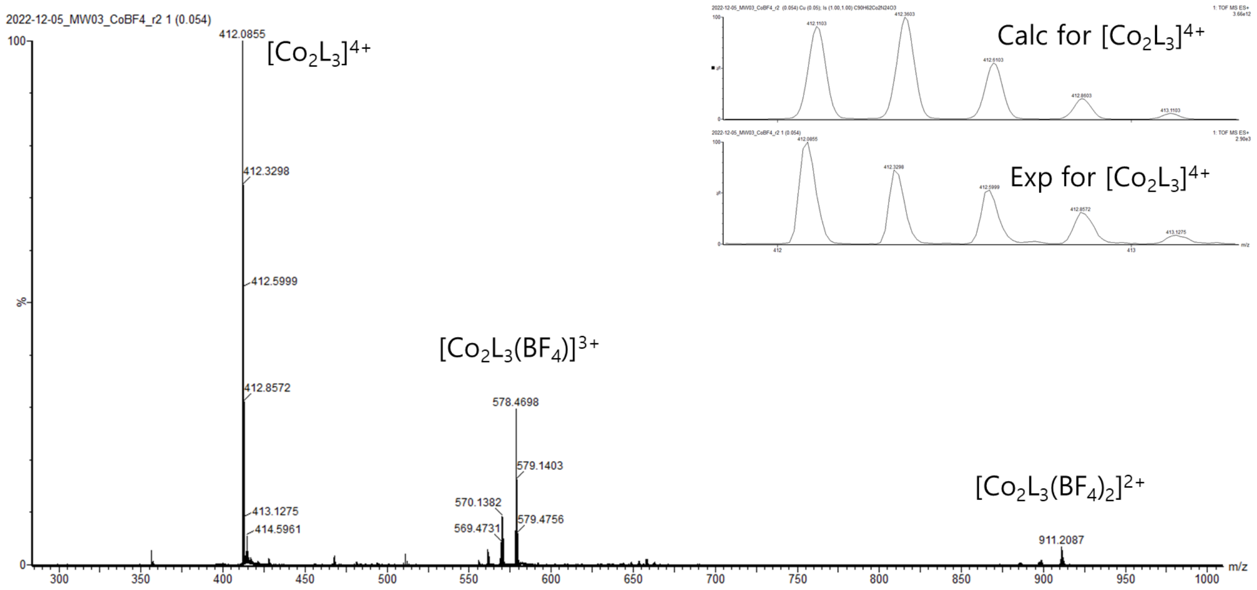

Figure S5. HR ESI-MS spectra for $[Co_2\mathbf{L}_3](BF_4)_4$ in acetonitrile. Inset shows the isotopic pattern for $[Co_2\mathbf{L}_3]^{4+}$ (bottom) with simulated pattern (top).

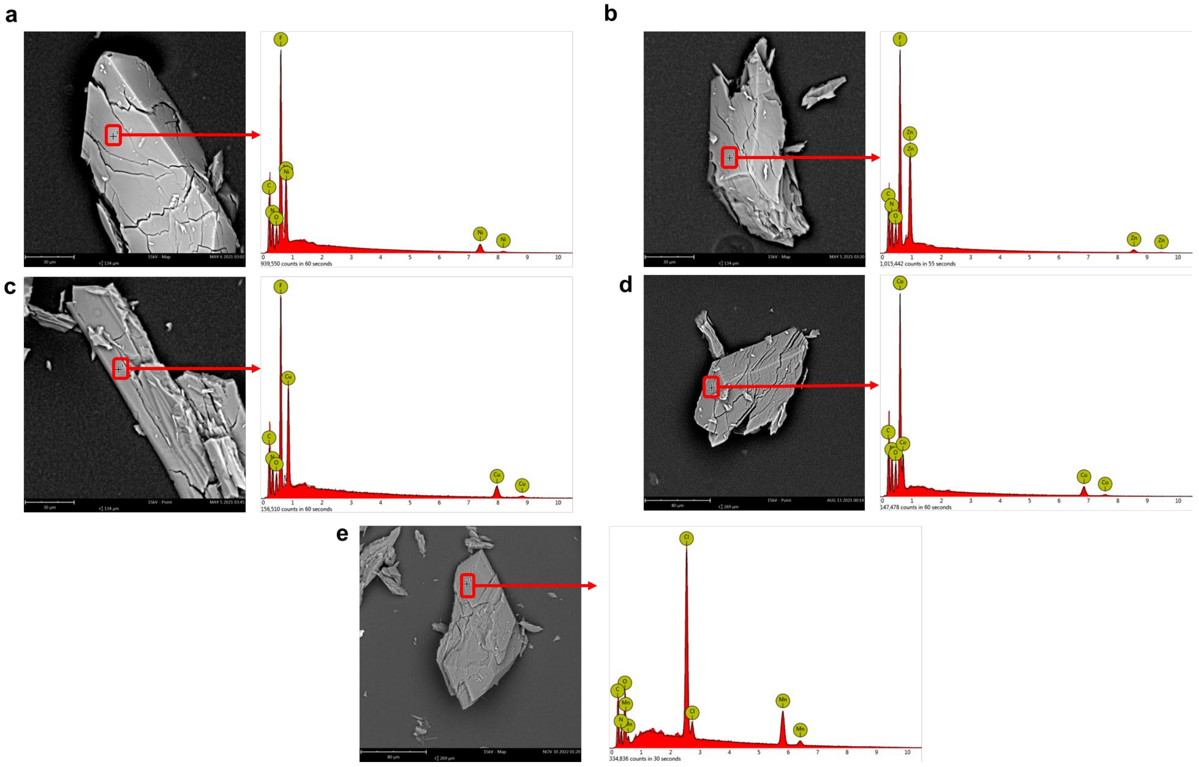

Figure S6. A backscattering SEM image (left) and corresponding EDS analysis (right) for a) $[Ni_2\mathbf{L}_3](BF_4)_4$; b) $[Zn_2\mathbf{L}_3](BF_4)_4$; c) $[Cu_2\mathbf{L}_3](BF_4)_4$; d) $[Co_2\mathbf{L}_3](BF_4)_4$ and e) $[Mn_2\mathbf{L}_3](ClO_4)_4$.

S2. Crystallographic data table for $[Mn_2\mathbf{L}_3](ClO_4)_4$, $[Co_2\mathbf{L}_3](BF_4)_4$, $[Ni_2\mathbf{L}_3](BF_4)_4$, $[Cu_2\mathbf{L}_3](BF_4)_4$ and $[Zn_2\mathbf{L}_3](BF_4)_4$.

Table S1. Crystallographic data table for compounds presented in this study.

Compound	$[Mn_2\mathbf{L}_3](ClO_4)_4$	$[Co_2\mathbf{L}_3](BF_4)_4$	$[Ni_2\mathbf{L}_3](BF_4)_4$	$[Cu_2\mathbf{L}_3](BF_4)_4$	$[Zn_2\mathbf{L}_3](BF_4)_4$
Identification code	2224285	2224286	2224287	2224286	2224289
Empirical formula	$C_{105}H_{75.5}Cl_4Mn_2N_{28.5}O_{20.5}$	$C_{102.34}H_{75}B_4Co_2F_{16}N_{27.5}O_{4.34}$	$C_{101}H_{70}B_4F_{16}N_{27.5}Ni_2O_{4.25}$	$C_{104.78}H_{90.28}B_4Cu_2F_{16}N_{29.5}O_{4.11}$	$C_{102}H_{84}B_4F_{16}N_{28}O_{4.25}Zn_2$
Formula weight	2316.11	2224.37	2201.50	2302.78	2247.95
Temperature (K)	100	100.00	100	100	100
Crystal system	Triclinic	triclinic	triclinic	triclinic	triclinic
Space group	*P-1*	*P-1*	*P-1*	*P-1*	*P-1*
a (Å)	16.060(3)	16.060(3)	16.230(3)	19.950(4)	16.120(3)
b (Å)	18.190(4)	17.710(4)	17.690(4)	20.310(4)	17.850(4)
c (Å)	20.480(4)	20.570(4)	20.430(4)	40.550(8)	20.560(4)
α (°)	79.38(3)	80.35(3)	80.45(3)	91.68(3)	80.07(3)

β (°)	88.85(3)	89.76(3)	89.74(3)	99.57(3)	89.62(3)
γ (°)	73.39(3)	72.80(3)	72.24(3)	94.23(3)	72.86(3)
Volume (Å^3)	5632(2)	5503(2)	5502(2)	16143(6)	5562(2)
Z	2	2	2	6	2
ρ_{calc} (g/cm^3)	1.366	1.342	1.329	1.421	1.342
μ (mm^{-1})	0.399	0.392	0.432	0.490	0.521
F(000)	2374.0	2268.0	2245.0	7082.0	2300.0
Crystal size (mm^3)	0.2 × 0.2 × 0.02	0.3 × 0.2 × 0.02	0.2 × 0.1 × 0.02	0.4 × 0.2 × 0.1	0.2 × 0.2 × 0.02
Radiation	MoKα (λ = 0.71073)	MoKα (λ = 0.71073)	MoKα (λ = 0.71073)	MoKα (λ = 0.71073)	MoKα (λ = 0.71073)
2Θ range for data collection (°)	2.024 to 49.424	2.01 to 50.7	2.024 to 52.044	2.012 to 52.044	2.014 to 43.934
Index ranges	-18 ≤ h ≤ 18, -21 ≤ k ≤ 21, -24 ≤ l ≤ 24	-19 ≤ h ≤ 19, -21 ≤ k ≤ 21, -24 ≤ l ≤ 24	-20 ≤ h ≤ 20, -21 ≤ k ≤ 21, -25 ≤ l ≤ 25	-24 ≤ h ≤ 24, -24 ≤ k ≤ 24, -50 ≤ l ≤ 50	-16 ≤ h ≤ 16, -18 ≤ k ≤ 18, -21 ≤ l ≤ 21

Reflections collected	121904	123589	127821	380367	88838
Independent reflections	18961 [R_{int} = 0.0277, R_{sigma} = 0.0169]	19708 [R_{int} = 0.0328, R_{sigma} = 0.0205]	20663 [R_{int} = 0.0280, R_{sigma} = 0.0181]	60876 [R_{int} = 0.0378, R_{sigma} = 0.0231]	13475 [R_{int} = 0.0478, R_{sigma} = 0.0283]
Data/restraints/parameters	18961/1480/1862	19708/1021/1727	20663/912/1435	60876/774/4527	13475/1093/1632
Goodness-of-fit on F^2	1.671	1.044	1.725	1.021	1.397
Final R indexes [I>=2σ (I)]	R_1 = 0.1100, wR_2 = 0.3512	R_1 = 0.0914, wR_2 = 0.2741	R_1 = 0.1109, wR_2 = 0.3683	R_1 = 0.0601, wR_2 = 0.1739	R_1 = 0.0998, wR_2 = 0.3043
Final R indexes [all data]	R_1 = 0.1257, wR_2 = 0.3776	R_1 = 0.1052, wR_2 = 0.2925	R_1 = 0.1239, wR_2 = 0.3899	R_1 = 0.0692, wR_2 = 0.1838	R_1 = 0.1255, wR_2 = 0.3389
Largest diff. peak/hole / e Å^{-3}	1.32/-0.96	1.17/-0.80	1.33/-0.81	1.72/-0.91	1.03/-0.81

S3. Crystallographic parameters for $[Mn_2\mathbf{L}_3](ClO_4)_4$, $[Co_2\mathbf{L}_3](BF_4)_4$, $[Ni_2\mathbf{L}_3](BF_4)_4$, $[Cu_2\mathbf{L}_3](BF_4)_4$ and $[Zn_2\mathbf{L}_3](BF_4)_4$.

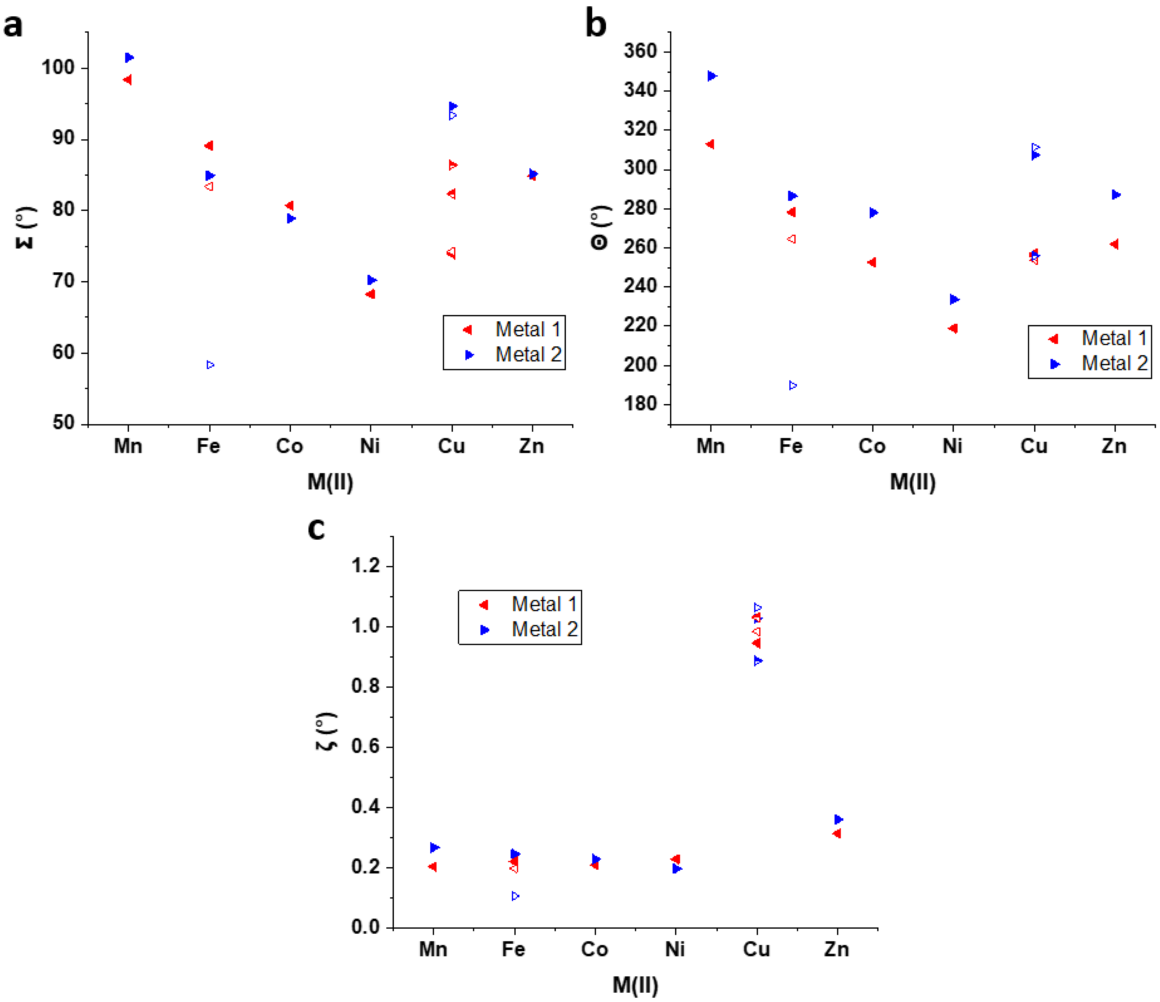

Figure S7. Octahedral distortion parameters. a) Σ; b) Θ; c) ζ. Solid shapes are used to represent [HS-HS] $[Fe_2\mathbf{L}_3](BF_4)_4$ and *Cu1-2* of $[Cu_2\mathbf{L}_3](BF_4)_4$, hollow shapes refer to [HS-LS] $[Fe_2\mathbf{L}_3](BF_4)_4$ and *Cu3-4* of $[Cu_2\mathbf{L}_3](BF_4)_4$, and half-coloured shapes refer to *Cu5-6* of $[Cu_2\mathbf{L}_3](BF_4)_4$.

S4. Chelate ring angles

Calculation of Pitch and Yaw

The angle between the chelate ring and intermetallic axis can be deconstructed into two components, representing the back and forth rocking and side to side twisting of each chelate ring, relative to the intermetallic axis. In order to quantify these motions, we adapted the concepts of pitch, roll and yaw, such that the combination of each angular component uniquely described the orientation of each chelate ring relative to the intermetallic axis.

Since we are using a simplistic model, involving only the coordinating N atoms, we assume that the roll component is negligible, and we thus define it to be zero. After taking the coordinates of Fei, Fej, Ni and Nj, we subject them to the following procedure to calculate the pitch and yaw angular components.

- All coordinates were translated such that Fei occupied the origin.
- The cross product of vectors Ni and Nj was calculated.
- All coordinates rotated about the x axis, such that the cross product lay in the xz axis.
- All coordinates were rotated about the y axis, such that the cross product lay on the z axis. This in turn means that Ni and Nj both lie in the xy plane.
- All coordinates were rotated about the z axis, such that Fej lay in the xz plane.
- Yaw was calculated using the Fej z and x coordinates by arctan(z/x).
- Pitch was calculated using the gradient of the Ni - Nj interval by arctan(Nj y - Ni y) / (Nj x - Ni x).

The signs of all yaw values were taken as positive, and the signs of pitch values were checked using the ratios of the y coordinates of Nj and Ni to determine the sign. Positive pitch was taken to mean the coordinating N in the imidazole lay closer to the intermetallic axis than the coordinating N of the imine, and the opposite is true in the case of negative pitch.

For more detailed explanations, please check references [1-3] in this supporting information.

S5. Pitch, yaw and bonding axes table for $[Mn_2\mathbf{L}_3](ClO_4)_4$, $[Co_2\mathbf{L}_3](BF_4)_4$, $[Ni_2\mathbf{L}_3](BF_4)_4$, $[Cu_2\mathbf{L}_3](BF_4)_4$ and $[Zn_2\mathbf{L}_3](BF_4)_4$.

Table S2. Selected geometric parameters describing structural orientation information for the dinuclear triple helicates.

Compound	M1 Secondary Bond Axis (˚)	M2 Secondary Bond Axis (˚)	M1 Mutual Bond Axis (˚)	M2 Mutual Bond Axis (˚)	M1 Pitch (˚)	M2 Pitch (˚)	M1 Yaw (˚)	M2 Yaw (˚)
$[Mn_2\mathbf{L}_3](ClO_4)_4$	**A**: 43.5, **B**: 46.4, **C**: 60.0	**A**: 55.0, **B**: 45.5, **C**: 39.8	**A-B**: 81.5, **A-C**: 85.1, **B-C**: 80.8	**A-B**: 84.7, **A-C**: 79.5, **B-C**: 75.3	**A**: 1.1, **B**: 1.3, **C**: 0.8	**A**: 1.0, **B**: 0.9, **C**: 1.3	**A**: 31.9, **B**: 33.2, **C**: 32.0	**A**: 36.0, **B**: 33.7, **C**: 30.4
$[Fe_2\mathbf{L}_3](BF_4)_4$ [HS-LS] [3]	**A**: 44.0, **B**: 32.1, **C**: 49.9	**A**: 44.2, **B**: 46.1, **C**: 32.7	**A-B**: 59.0, **A-C**: 83.9, **B-C**: 71.5	**A-B**: 72.1, **A-C**: 66.1, **B-C**: 69.2	**A**: 7.0, **B**: 4.9, **C**: -3.5	**A**: -2.9, **B**: -0.8, **C**: 8.4	**A**: 33.3, **B**: 34.2, **C**: 31.5	**A**: 33.1, **B**: 37.9, **C**: 32.5
$[Fe_2\mathbf{L}_3](BF_4)_4$ [HS-HS] [3]	**A**: 43.5, **B**: 43.6, **C**: 52.4	**A**: 54.3, **B**: 52.4, **C**: 40.9	**A-B**: 78.7, **A-C**: 78.4, **B-C**: 75.0	**A-B**: 85.9, **A-C**: 78.7, **B-C**: 73.8	**A**: 2.3, **B**: 8.6, **C**: -6.6	**A**: 2.6, **B**: -4.1, **C**: 6.7	**A**: 31.7, **B**: 33.4, **C**: 33.4	**A**: 35.6, **B**: 34.0, **C**: 33.1
$[Co_2\mathbf{L}_3](BF_4)_4$	**A**: 42.7, **B**: 40.9, **C**: 54.9	**A**: 52.7, **B**: 52.2, **C**: 37.9	**A-B**: 74.8, **A-C**: 81.9, **B-C**: 74.2	**A-B**: 88.3, **A-C**: 75.5, **B-C**: 72.8	**A**: 1.1, **B**: 1.2, **C**: 0.9	**A**: 1.0, **B**: 0.9, **C**: 1.3	**A**: 32.8, **B**: 34.5, **C**: 33.3	**A**: 36.0, **B**: 35.3, **C**: 32.6
$[Ni_2\mathbf{L}_3](BF_4)_4$	**A**: 41.3, **B**: 40.5, **C**: 51.0	**A**: 50.9, **B**: 49.7, **C**: 39.0	**A-B**: 72.0, **A-C**: 77.0, **B-C**: 73.8	**A-B**: 88.1, **A-C**: 75.0, **B-C**: 71.3	**A**: 1.1, **B**: 1.2, **C**: 0.8	**A**: 1.1, **B**: 0.9, **C**: 1.2	**A**: 32.4, **B**: 34.2, **C**: 33.9	**A**: 34.7, **B**: 35.7, **C**: 34.1
$[Cu_2\mathbf{L}_3](BF_4)_4$ (*Cu1-2*)	**A**: 38.2, **B**: 43.4, **C**: 56.5	**A**: 54.1. **B**: 45.6, **C**: 39.6	**A-B**: 66.4, **A-C**: 88.3, **B-C**: 75.1	**A-B**: 84.8, **A-C**: 76.5, **B-C**: 72.4	**A**: 1.1, **B**: 1.2, **C**: 0.9	**A**: 1.1, **B**: 0.9, **C**: 1.2	**A**: 35.3, **B**: 36.8, **C**: 24.8	**A**: 36.9, **B**: 35.9, **C**: 29.5
$[Cu_2\mathbf{L}_3](BF_4)_4$ (*Cu3-4*)	**D**: 36.9, **E**: 44.0, **F**: 50.6	**D**: 55.8, **E**: 47.3, **F**: 42.5	**D-E**: 73.7, **D-F**: 79.4, **E-F**: 63.4	**D-E**: 90.0, **D-F**: 80.4, **E-F**: 73.1	**D**: 1.2, **E**: 1.3, **F**: 0.8	**D**: 1.2, **E**: 0.8, **F**: 1.2	**D**: 33.1, **E**: 34.0, **F**: 30.3	**D**: 37.4, **E**: 36.4, **F**: 30.4
$[Cu_2\mathbf{L}_3](BF_4)_4$ (*Cu5-6*)	**G**: 41.1. **H**: 32.2. **I**: 55.7	**G**: 53.5, **H**: 41.8, **I**: 39.2	**G-H**: 65.7, **G-I**: 69.3, **H-I**: 79.4	**G-H**: 79.8, **G-I**: 71.5, **H-I**: 73.5	**G**: 1.0, **H**: 1.3, **I**: 1.0	**G**: 1.1, **H**: 1.0, **I**: 1.1	**G**: 32.7, **H**: 33.9, **I**: 34.1	**G**: 36.8, **H**: 34.7, **I**: 31.8
$[Zn_2\mathbf{L}_3](BF_4)_4$	**A**: 42.8, **B**: 41.3, **C**: 50.8	**A**: 53.5, **B**: 52.9, **C**: 38.6	**A-B**: 76.3, **A-C**: 76.0, **B-C**: 72.7	**A-B**: 86.7, **A-C**: 75.8, **B-C**: 74.1	**A**: 1.1, **B**: 1.3, **C**: 0.8	**A**: 1.1, **B**: 0.9, **C**: 1.2	**A**: 31.8, **B**: 33.5, **C**: 32.7	**A**: 35.6, **B**: 34.3, **C**: 31.7

S6. References

1. Min, H.; Craze, A.R.; Wallis, M.J.; Tokunaga, R.; Taira, T.; Hirai, Y.; Bhadbhade, M.M.; Fanna, D.J.; Marjo, C.E.; Hayami, S.; Lindoy, L.F.; Li, F. Spin crossover induced by changing the identity of the secondary metal species in a face-centred $FeII_8NiII_6$ cubic cage. *Chem. Eur. J.* **2022**, https://doi.org/10.1002/chem.202203742
2. Min, H.; Craze, A.R.; Taira, T.; Wallis, M.J.; Bhadbhade, M.M.; Tian, R.; Fanna, D.J.; Wuhrer, R.; Hayami, S.; Clegg, J.K.; Marjo, C.E.; Lindoy, L.F.; Li, F. Self-Assembly of a Rare High Spin FeII/PdII Tetradecanuclear Cubic Cage Constructed via the Metalloligand Approach. *Chemistry.* **2022**, *4*, 535-547.
3. Wallis, M.J.; Craze, A.R.; Zenno, H.; Tokunaga, R.; Taira, T.; Min, H.; Bhadbhade, M.M.; Bhattacharyya, S.K.; Tian, R.; Rich, A.; Hayami, S.; Clegg, J.K.; Marjo, C.E.; Lindoy, L.F.; Li, F. Unique spin crossover pathways differentiated by scan rate in a new dinuclear Fe(II) triple helicate: Mechanistic deductions enabled by synchrotron radiation studies. *ChemRxiv.* https://doi.org/10.26434/chemrxiv-2022-4hw9h
